# Seasonal variability in the feeding ecology of an oceanic predator

**DOI:** 10.1038/s41598-024-63557-z

**Published:** 2024-07-29

**Authors:** Mitchell S. Lovell, Michael J. Polito, Josef A. Schuster, Emily E. Shallow, Alexis M. Janosik, Brett J. Falterman, Michael A. Dance

**Affiliations:** 1 Department of Oceanography & Coastal Sciences, Louisiana State University, Baton Rouge, LA 70803 USA; 2 Department of Biology, University of West Florida, Pensacola, FL 32514 USA; 3 Fisheries Research Support LLC, Mandeville, LA 70448 USA

**Keywords:** Behavioural ecology, Biooceanography, Ecosystem ecology, Stable isotope analysis

## Abstract

Complementary approaches (stomach contents, DNA barcoding, and stable isotopes) were used to examine seasonal shifts in the feeding ecology of an oceanic predator, yellowfin tuna (*Thunnus albacares*, n = 577), in the northern Gulf of Mexico. DNA barcoding greatly enhanced dietary resolution and seasonally distinct prey assemblages were observed for both sub-adults and adults. In general, diet was characterized by ommastrephid squids and exocoetids in spring, juvenile fishes (i.e., carangids and scombrids) in summer, migratory coastal fishes during fall, and an increased consumption of planktonic prey (e.g., amphipods) in winter. Seasonal variability in bulk stable isotope values (δ^13^C, δ^15^N, and δ^34^S) was also observed, with low δ^15^N values and high δ^34^S values during late summer/early fall and high δ^15^N values (low δ^34^S) during late winter/early spring. Bayesian stable isotope mixing models corroborated seasonal diet shifts, highlighting the importance of oceanic nekton in spring/summer, coastal nekton during fall, and oceanic plankton during winter. Seasonal shifts in diet appeared to be influenced by prey reproductive cycles, habitat associations, and environmental conditions. Findings highlight the complex food web dynamics supporting an opportunistic oceanic predator and the importance of seasonal cycles in prey availability to predator resource utilization in open-ocean ecosystems.

## Introduction

Large predators play significant roles in structuring open-ocean ecosystems^1^ via top-down control^2,3^ and are capable of basin-scale movements that ecologically link distinct regions and ecosystems^1,4^. Over the past few decades, populations of many oceanic predators have declined due to various anthropogenic impacts including overfishing and habitat loss/alteration^5,6^. Because of this, an improved understanding of the drivers which sustain oceanic predator populations, contribute to habitat quality, and influence movement/distribution is needed to effectively conserve essential habitats and food webs critical to maintaining open-ocean ecosystems^7^.

Characterizing the diet of predators provides valuable information on foraging behavior, requisite prey assemblages, and the complex dynamics that support their populations^8^. While some predators are specialists and feed on specific prey^9^, many taxa are generalists and opportunistically utilize a wider range of prey resources^10^. As a result, fluctuations in prey availability can lead to temporal shifts in the diets of opportunistic predators as they exploit seasonally abundant and/or available resources^11–14^. This is particularly true in open-ocean ecosystems, where the dynamic nature of physical and biological processes leads to spatial and temporal variability in habitat and prey availability^15^. Unfortunately, dietary studies are often limited in temporal resolution and lack the level of repeated sampling needed to characterize diet shifts across the seasonal cycle^16^. Thus, despite the importance of oceanic predators in structuring open-ocean food webs, our understanding of seasonal foraging dynamics and the suite of prey resources needed to support their populations remains limited^17^.

Here, complementary analyses of stomach contents, DNA barcoding, and stable isotopes were used to characterize seasonal variability in the feeding ecology of a model oceanic predator, yellowfin tuna (*Thunnus albacares*). Yellowfin tuna are circumtropical oceanic predators that also inhabit marginal seas such as the Gulf of Mexico, and are of considerable ecological and economic value^1,15,18–20^. The northern Gulf of Mexico (nGoM) is characterized by a temporally dynamic convergence of nutrient-rich water from the Mississippi River, strong oceanic currents from the Loop Current (and associated eddies), and unique habitat features (i.e., oil and gas platforms^21^) that provide ideal habitat for both foraging and spawning yellowfin tuna^1,22–24^. However, our understanding of the prey resources and trophic interactions that support these populations remains limited^25,26^. The aim of this study was to examine variability in yellowfin tuna diet at a high temporal resolution, while employing DNA barcoding to increase the taxonomic resolution of the consumed prey assemblage. Specifically, our goal was to characterize seasonal shifts in prey resources used by sub-adult and adult yellowfin tuna in the nGoM using stomach contents (short-term feeding: hours), stable isotope analysis of δ^13^C, δ^15^N, and δ^34^S (long-term feeding: months), and Bayesian stable isotope mixing models.

## Methods

### Sample collection

Yellowfin tuna were sampled once a week in the nGoM over a one-year period (April 2019–March 2020) to capture potential seasonal variability in their feeding ecology. Samples were obtained opportunistically from recreational charter landings in Venice, Louisiana (rod-and-reel fishery) via dockside sampling. Although the exact time and location of capture for each specimen was not recorded, a general area of capture was estimated through conversations with the charter captains (Supplemental Fig. S1). Yellowfin tuna were caught from various habitats in both oceanic and coastal ecosystems, primarily around offshore petroleum infrastructure such as fixed platforms, floating platforms (e.g., tension leg, SPAR), and drill ships; hereafter, collectively referred to as oil and gas platforms^27^. Individuals were measured to the nearest 0.5 cm fork length and sexed before removing the stomach and excising a 5 cm^3^ section of white muscle tissue from the dorsal region. Because the recreational charter fishery in the nGoM frequently uses live bait and/or chum (i.e., cut pieces of bait or chunks of fish carcasses) to capture yellowfin tuna, the usage of bait and chum was recorded for each individual via conversations with the charter captains. All samples were stored on ice (< 6 h) and transported to the laboratory, where both white muscle tissue and stomachs were stored at − 20 °C until later processing.

### Stomach content analysis

Yellowfin tuna stomachs were thawed, opened, and all contents were sorted. Any contents identified as bait or chum were discarded from further analyses. Prey items were identified as bait when accompanied with obvious hook/knife marks or when reported as being used as bait by the charter captain, whereas chum was exclusively cut pieces of muscle tissue (often from tuna carcasses) or cut bait. The remaining items were considered natural prey and were identified to the lowest possible taxonomic grouping. Prey items were blotted with Kimtech Kimwipes to remove excess water and then weighed to the nearest 0.1 g^28^. The approximate life-stage of prey items was estimated based on their relative size and knowledge of their life-history; however, prey length and girth was not recorded. Furthermore, tissues from several prey specimens were also sampled in preparation for stable isotope analysis to assess the contribution of various prey sources to yellowfin tuna diets. A small sample of muscle tissue was excised from fishes (epaxial) and squids (mantle), while crustaceans (solely amphipods) were sampled whole. Prey items selected for stable isotope analysis were restricted to high-quality specimens, where little or no digestion was observed. These samples were stored at − 20 °C for subsequent stable isotope analysis.

DNA barcoding was utilized to identify prey items that otherwise could only be assigned to broad taxonomic groupings (e.g., unidentified fishes, squids, crustaceans, etc.) due to advanced stages of digestion in the stomach. A small section (1 cm^3^) of muscle tissue was excised from these individuals, rinsed with deionized water to avoid/limit cross-contamination, preserved in 95% ethyl alcohol, and then shipped to the Janosik Lab at the University of West Florida. DNA of prey samples were extracted using a DNeasy Blood and Tissue Kit (Qiagen®, Hilden, Germany), where a 655-base pair region of the mitochondrial COI gene was amplified using FishF2 and FishR2 primers^29^. Purified PCR products were then visualized on a 1.5% agarose gel stained with ethidium bromide. Positive PCR reactions were purified using Exonuclease I and Fast Alkaline Phosphatase (ExoFAP, ThermoFisher Scientific), bi-directionally sequenced by Arizona Research Laboratories (Tucson, AZ), edited in Sequencher version 5.4.3 (Gene Codes Corporation), and checked against the BLAST nucleotide database (https://blast.ncbi.nlm.nih.gov/Blast.cgi) to confirm species-level identity^30^.

Yellowfin tuna were examined across two size classes to account for the influence of body size on their diet^31^. Size classes, representing approximate life stages, consisted of sub-adults (< 100 cm) and adults (> 100 cm)^32,33^. Empty stomachs and those containing only parasites, *Sargassum* sp., and/or unidentified prey were not used for statistical comparisons. For the purposes of this study, seasons were defined as spring (April–June), summer (July–September), fall (October–December), and winter (January–March) to best represent changing air and water temperatures in the nGoM. Prey items that did not achieve family-level (or more precise) taxonomic classification via morphological identification or during DNA barcoding were omitted from statistical analysis (sub-adult and adult yellowfin tuna averaged 4.2 and 3.3 unidentified prey items per stomach, respectively). Percent composition by number (%N), percent composition by wet weight (%W), and percent frequency of occurrence (%FO) were calculated at both the lowest identifiable taxonomic rank and family-level for each strata examined: size class and season. An index of relative importance (%IRI), using both weight and numerical-based metrics of prey contribution, was then calculated from %N, %W, and %FO (1) and used to evaluate prey importance within each season for each size class^28^.

1 $$\begin{aligned}\text{IRI }= \, &\left(\%\text{N }+ \, \%{\text{W}}\right) \, \times \, \%{\text{FO}} \\ &\text{and} \\\text{\%IRI }=&\left(\frac{\text{IRI}}{\Sigma \text{ IRI}}\right)\times \text{ 100} \end{aligned}$$

All statistical tests were performed in the R Statistical Programming Environment (Version 4.0.5)^34^ using an alpha value of 0.05. Species accumulation curves were constructed using the vegan package in R^35^ and qualitatively assessed to determine if the sample size per season and size class was adequate in describing seasonal shifts in yellowfin tuna diets^35^. Seasonal patterns in beta diversity of prey assemblages were distinguished using permutational analysis of variance (PERMANOVA). To statistically assess differences in prey community composition, a weighted metric accounting for both percent composition by number and biomass of prey taxa was developed, where Eq. (2):

2 $$\text{\%NW = }\frac{\text{ (\%N + \%W)}}{2}$$

was calculated for each prey taxa per individual stomach and then used to create a Bray–Curtis dissimilarity matrix. A PERMANOVA was performed on the matrix to evaluate the effects of season on prey composition for sub-adult and adult yellowfin tuna using the vegan package^35^. To identify seasonal differences in the diets, pairwise comparisons were examined using the pairwise.adonis2() function developed by Martínez Arbizu^36^. Lastly, seasonal patterns in the occurrence of prey taxa that were frequently observed in the overall diet of yellowfin tuna (sub-adult and adult diets combined) were examined and visualized using generalized additive models (GAMs). Prey occurrence (1 = present, 0 = absent) was modeled against day of year (1–366, where day 1 was April 1st) and fitted with a binomial distribution using a logit link in the R package mgcv. Cyclic cubic regression splines were penalized from a specified maximum basis dimension (k = 6) with the degree of each penalty and smoothness automatically selected by restricted maximum likelihood (REML).

### Stable isotope analysis

In dietary studies, δ^15^N values are often used to assess relative trophic position^24,37^, while δ^34^S values are used to contrast contribution of benthic/pelagic and/or freshwater/marine sources to a consumer’s diet^38,39^. δ^13^C values are often used to examine basal sources of organic carbon in a consumer’s diet since ^13^C fractionates little between trophic steps^24,40^; however, given that the isotopic incorporation of organic carbon into a consumer’s tissue spans large temporal scales^31^, δ^13^C can also be used to assess long-term movement patterns^41^. Here, stable isotope analysis of δ^13^C, δ^15^N, and δ^34^S was performed on a subset of sub-adult (n = 120, 30 per season) and adult (n = 120, 30 per season) yellowfin tuna and select prey items (n = 50; excised during stomach content analysis) at the Louisiana State University Stable Isotope Ecology Laboratory. To minimize the influence of body size on stable isotope values, yellowfin tuna samples were systematically chosen to obtain a comparable fork length among seasons for each size class. Prior to analysis, white muscle tissue of yellowfin tuna and prey items were freeze-dried for 48 h and then homogenized using a mortar and pestle. Each homogenized tissue sample (1.5 ± 0.025 mg), accompanied with 3.0 ± 0.025 mg of vanadium pentoxide (a catalyst used in sulfur isotope analysis)^42^, was loaded into a 5 × 9-mm tin capsule for stable isotope analysis using an EA IsoLink IRMS System (Thermo Scientific) interfaced with a Delta V Advantage continuous flow isotope ratio mass spectrometer (Thermo-Fisher). Stable isotope values were normalized using a two-point system with glutamic acid reference materials (IAEA-S2 and IAES-S3) for sulfur. Stable isotope values were expressed in delta notation (δ) and per mil units (‰), relative to international measurement standards Vienna Pee Dee Belemnite (carbon), atmospheric N_2_ (nitrogen), and Vienna Canyon Diablo troilite (sulfur), using Eq. (3), where R represents the ratio of heavy to light isotopes (^13^C/^12^C, ^15^N/^14^N, ^34^S/^32^S). Sample precision was ± 0.1‰ for δ^13^C, ± 0.2‰ for δ^15^N, and ± 0.3‰ for δ^34^S based on repeated analysis of reference materials (i.e., USGS-40, USGS-41, IAEA-S-2, IAEA-S-3, and Louisiana State University Stable Isotope Ecology Laboratory’s *Sciaenops ocellatus* muscle tissue).

3 $${\updelta }^{13}\text{C, }{\updelta }^{15}\text{N, or } {\updelta }^{34}{\text{S }} \left(\permil \right) = \left(\frac{{\text{R}}_{\text{sample}}}{{\text{R}}_{\text{standard}}} -\text{ 1}\right)\times \text{ 1000}$$

Because high lipid content in tissues can lead to inaccurate interpretations of δ^13^C values^43^, a species-specific arithmetic lipid correction equation [Eq. (4)] was used and applied to yellowfin tuna white muscle tissue samples with C:N ratios that exceeded 3.14 (x-intercept)^43^. Similarly, lipid correction equations referenced from the best available literature (Supplemental Table S1)^44–47^ were applied to prey tissue samples with C:N ratios greater than 3.5^44^.

4 $${\updelta }^{13}{{\text{C}}}_{\text{corrected}}\text{ = }{\updelta }^{13}{{\text{C}}}_{\text{untreated }}+ \frac{\text{(}\left(\text{9.356 }\times \text{ C:N}\right) -\text{ 29.359)}}{\text{(C:N }+\text{ 2.181)}}$$

The response of δ^13^C, δ^15^N, and δ^34^S values to season and size class were examined using generalized additive mixed models (GAMMs). Modeling followed a hierarchical generalized additive model (HGAM) framework as described by Pedersen et al.^48^, which is helpful in identifying ecological patterns (i.e., seasonality) and differences between groups (i.e., size classes). Models were developed for each isotopic ratio with isotope value as the response variable, day of year (1–366, day 1 = April 1st) as the explanatory variable, and size class included as a random effect (5).

5 $${\text{E}}\left(\text{Stable \, Isotope \, Value}\right)\text{ = f}\left(\text{Day \, of \, Year}\right)\text{ + }\upzeta \left(\text{Size \, Class}\right)\text{ + }{\upvarepsilon }_{\text{i}}$$

Hierarchical generalized additive models were developed to allow the smooth term for each random effect (i.e., Size Class) to vary independently in both shape and wiggliness (model I)^48^. Smoothing parameters were selected using REML since it produces less variability when smoothing and is more resistant to overfitting data^49^. Models were fitted with a Gaussian distribution using the mgcv package in R^50^, in which cyclic cubic regression splines were penalized from a basis dimension (k) of 8 for all models to avoid overfitting and unrealistic ecological interpretations^51^.

### Bayesian stable isotope mixed modeling

The relative contribution of prey sources to the diets of sub-adult and adult yellowfin tuna in each season was estimated with Bayesian stable isotope mixing models (BSIMMs) using the MixSIAR package in R^52^. BSIMMs incorporate predator stable isotope values (i.e., the consumer), prey stable isotope values (i.e., the sources), trophic discrimination factors (TDFs), and also allow for the integration of prior information (often stomach content data or fecal matter). Priors inform BSIMMs on the expected proportional contribution of a source to a consumer’s diet and ultimately improve model performance^52^. In this study, informative priors were calculated from stomach content data, following the recommendations of Stock et al. (2018, Eq. 4)^52^. Additionally, to account for turnover rates in yellowfin tuna muscle tissue and to align the BSIMM results with stomach content data, a time lag of 6 and 9 months was applied to the isotope values of sub-adult and adult yellowfin tuna, respectively. This time lag estimate was based on previous turnover rate studies for tunas and allometric scaling of body size^24,31,41^.

Source data was obtained from stable isotope analyses of prey items found in the stomach contents of yellowfin tuna. Before performing BSIMMs, prey taxa were split into three source groups (coastal nekton, oceanic nekton, or oceanic plankton) based on a cluster analysis of their stable isotope values (Supplemental Table S2)^53^. For the purposes of this study, nekton refers to various fishes and squids from both oceanic and coastal ecosystems, while plankton refers to small-bodied prey, such as amphipods, gastropods, larval fishes, larval squids, and other gelatinous organisms. Stable isotope data was pooled for each of the three source groups to generate a specific mean, standard deviation (± SD), and concentration dependency (Supplemental Table S3). TDFs (Δ^13^C and Δ^15^N) used in BSIMMs were based on values reported from previous studies on tuna, where white muscle tissue TDFs and associated standard deviations (± SD) were estimated to be 0.82 ± 1.13 for δ^13^C (averaged δ^13^C TDF from Varela et al. and Madigan et al.)^40,54^ and 2.1 ± 1.0 for δ^15^N^55^. Due to higher relative support (lower LOOic score)^56^ for BSIMMs with δ^34^S excluded and the lack of comparable δ^34^S TDFs for tuna-like species, δ^34^S was not included in the final models. Priors were constructed for each source group in both sub-adult and adult BSIMMs from stomach content data using the averaged %NW (2) of prey taxa across season. Priors were incorporated into BSIMMs and estimated to be 0.06 (coastal nekton), 2.02 (oceanic nekton), and 0.91 (oceanic plankton) for the sub-adult BSIMM. The adult BSIMM priors were calculated to be 0.36 (coastal nekton), 2.32 (oceanic nekton), and 0.32 (oceanic plankton). Lastly, elemental concentration dependence^57^ was incorporated into both BSIMMs, as well as residual and process error, with each model run being 1 million iterations (500,000 burn-ins) and thinned by 500. Model convergence and fit were checked using Gelman-Rubin diagnostic values (i.e., Gelman–Rubin statistics < 1.1) and by plotting the posterior predictive distributions^58^.

## Results

### Stomach contents

A total of 371 sub-adult and 206 adult yellowfin tuna (64.5–183.5 cm fork length) were collected during the study period, of which 311 sub-adult and 178 adult stomachs were used in stomach content analyses to investigate seasonal variability among prey assemblages within each size class (Supplemental Table S4). Collectively, 114 unique prey taxa representing 60 families (and the order Stomatopoda, Supplemental Tables S5 and S6) were identified using taxonomic keys relevant to the nGoM^59–61^. Overall, yellowfin tuna consumed a diverse array of taxa including Actinopterygii (bony fishes), Crustacea (amphipods, crabs, lobsters, shrimps, and stomatopods), Cephalopoda (squids and octopus), Echinodermata (sea stars), Gastropoda (solely marine snails), and Tunicata (salps). A total of 43 samples were tested for DNA barcoding, of which 39 amplified successfully. Of the successful amplifications, 36 samples had BLAST query coverages (the percent of the query sequence that overlaps the reference sequence) greater than 95% (range 74–100%), while 35 of those samples had genetic matches greater than 96.6% (range 84.3–100%). After DNA barcoding, prey items that were not identified to at least family-level were reduced to 7% of the observed specimens.

Seasonal variability in prey assemblages (beta diversity) was observed for both sub-adult and adult yellowfin tuna (PERMANOVA; p < 0.001). Nearly all possible pairwise combinations of season and size class were statistically different (pairwiseAdonis; p < 0.01) with the exception of sub-adult and adult diets during the spring (p > 0.05). These differences were largely driven by nine prey taxa (Carangidae, Coryphaenidae, Exocoetidae, Nomeidae, Ommastrephidae, Phrosinidae, Scombridae, Serranidae, and Stomatopoda) which collectively accounted for greater than 50% of the dissimilarity in diet across all size class and seasonal comparisons (SIMPER; Supplemental Table S7).

**Figure 1:**
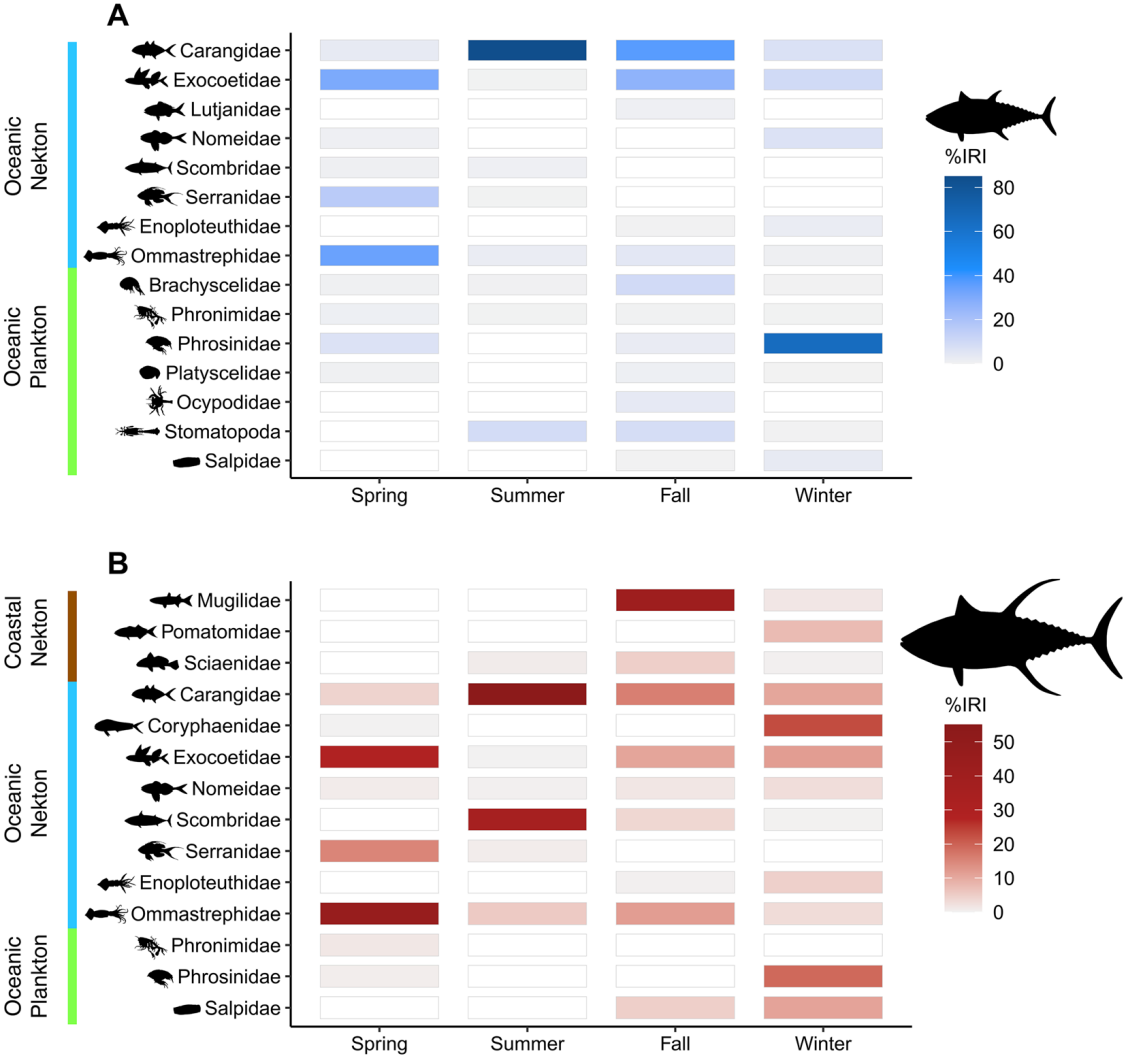
Heat matrices showing the seasonal contribution of prey taxa that contributed at least 1% to %IRI (within any given season) to the diets of sub-adult (**A**) and adult (**B**) yellowfin tuna from the northern Gulf of Mexico (nGoM). Note, the %IRI legend for sub-adults and adults are on different scales. White regions represent a contribution of 0%IRI to the diet, while colored regions (color scale of gray to blue or gray to red) represent a contribution greater than 0%IRI. Darker shades of blue (**A**) and red (**B**) signify a higher contribution to %IRI. On the y-axis, prey taxa were categorized as coastal nekton (brown), oceanic nekton (blue), or oceanic plankton (green).

Although a wide range of prey were identified in the stomach contents, the majority of diets were characterized by relatively few prey taxa (Fig. 1). Spring prey assemblages for both sub-adult and adult yellowfin tuna were largely represented by ommastrephid squids, exocoetids, and juvenile reef-associated fishes (e.g., serranids). Ommastrephid squids contributed the most to %IRI for both sub-adult (34.1%IRI) and adult (46.3%IRI) diets during spring, while exocoetids and serranids represented much of the remaining diet. Carangids (predominantly juveniles) were the most abundant prey taxa for both sub-adult (84.6%IRI) and adult (54.2%IRI) yellowfin tuna during summer, with blue runner (*Caranx crysos*) being the most frequently encountered species in both size classes. Other prominent prey taxa during summer included pelagic-oriented stomatopod larvae (9.2%IRI) for sub-adults and juvenile scombrids (35.7%IRI) for adults. Sub-adult diets during fall primarily consisted of carangids (36.9%IRI), exocoetids (26.7%IRI), and brachyscelid amphipods (9.8%IRI). In contrast, adult yellowfin tuna consumed coastal fishes, such as mugilids (41.1%IRI), as well as carangids (16.1%IRI), ommastrephid squids (11.9%IRI), and exocoetids (10.8%IRI). Finally, the winter prey assemblage of sub-adult yellowfin tuna was characterized by phrosinid amphipods (65.4%IRI) followed by exocoetids (9.9%IRI), carangids (7.1%IRI), and nomeids (6.5%IRI). Adult diets during winter were represented by juvenile coryphaenids (23.0%IRI), phrosinid amphipods (19.2%IRI), exocoetids (11.9%IRI), salps (11.0%IRI), and carangids (10.7%IRI).

**Figure 2:**
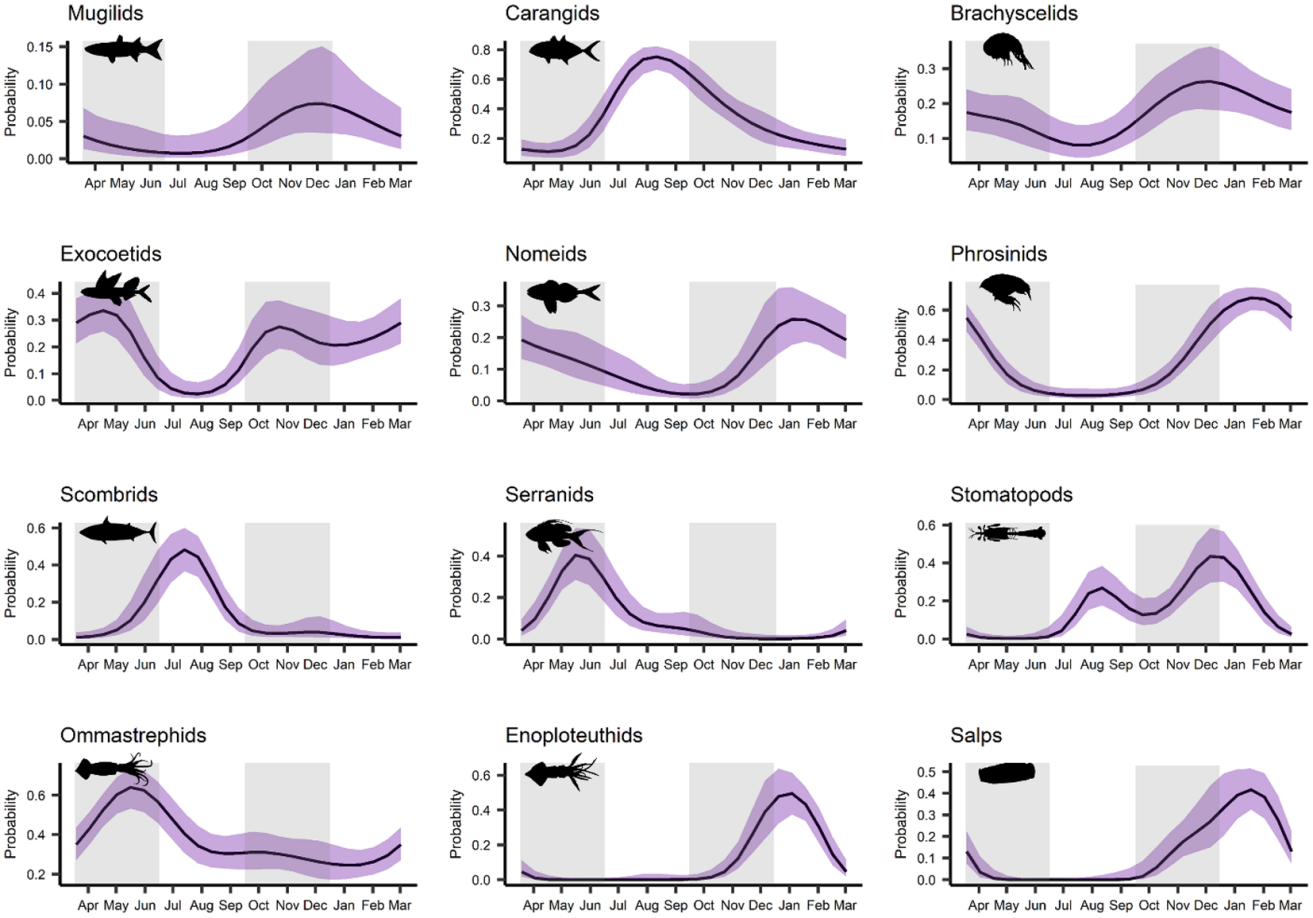
Response plots from generalized additive models (GAMs) showing seasonal trends in the probability of occurrence of prominent prey taxa in stomachs of yellowfin tuna in the northern Gulf of Mexico (nGoM). Shading (grey regions) separates the four seasons (spring, summer, fall, and winter). Note, y-axes (probability of occurrence) are on different scales.

The occurrence of prominent prey taxa in yellowfin tuna stomachs varied temporally, and significant seasonal trends were evident for each taxa examined (GAM; p < 0.05, Fig. 2, Supplemental Table S8). Strong seasonality was observed for several oceanic nekton, including serranids (spring), ommastrephid squids (spring), scombrids (summer), carangids (summer), enoploteuthid squids (winter), and nomeids (winter). Peak probability of occurrence for phrosinid amphipods, brachyscelid amphipods, and stomatopod larvae was observed during early winter; however, the probability of occurrence for many of these planktonic taxa was also high during spring and/or fall. In contrast, mugilids (a coastal-oriented fish) did not exhibit strong seasonality, but were most likely to be encountered in late fall. Exocoetids and larval stomatopods displayed bimodal trends, with peaks occurring in two separate seasons. The probability of occurrence for exocoetids was greatest during spring with a second peak in fall, while the probability of occurrence for stomatopod larvae was characterized by a stronger peak during late fall to early winter and a weaker peak during summer.

### Stable isotopes

**Figure 3:**
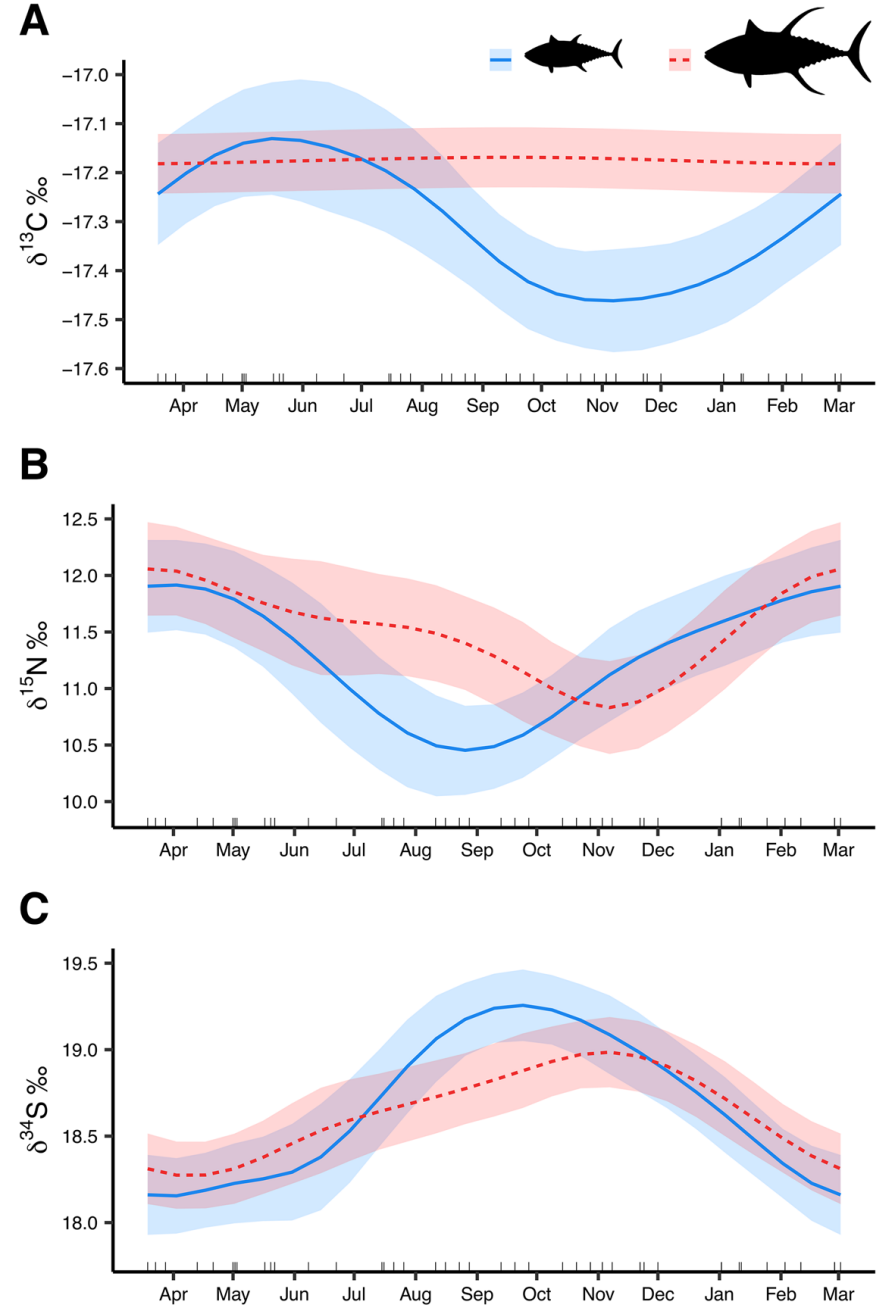
Response plots from hierarchical generalized additive mixed models showing the effect of day-of-year on δ^13^C (**A**), δ^15^N (**B**), and δ^34^S (**C**) values for sub-adult (blue) and adult (red) yellowfin tuna from the northern Gulf of Mexico (nGoM). Tick marks along the inside of the x-axis represent exact days from which the samples were collected. All seasonal trends were statistically significant (p < 0.05) with the exception of δ^13^C values for adult yellowfin tuna.

Seasonal patterns in δ^13^C, δ^15^N, and δ^34^S values were examined in both sub-adult and adult yellowfin tuna (Fig. 3). Sub-adult δ^13^C values ranged from − 18.3‰ to − 16.5‰, while adult δ^13^C values ranged from − 18.2‰ to − 16.1‰. Significant seasonal trends in δ^13^C values were observed for sub-adults (HGAM; p < 0.001, Supplemental Table S9), with values peaking during spring (− 17.1‰) and declining through fall (− 17.5‰). In contrast, no seasonal trend was detected for adult δ^13^C values (HGAM; p > 0.05, Supplemental Table S9). Overall, δ^15^N values for sub-adult yellowfin tuna ranged from 8.3 to 13.9‰, while adult values ranged from 7.6 to 14.7‰. Significant seasonal trends in δ^15^N values were evident for both size classes (HGAM; p < 0.05, Supplemental Table S9), with higher δ^15^N values observed for both size classes during late winter/early spring. Minimum δ^15^N values for sub-adults were observed during late summer (10.5‰), while adult δ^15^N values were lowest during late fall/early winter (10.8‰). The observed range of δ^34^S values were similar between size classes, where sub-adult values ranged from 16.5‰ to 20.0‰ and adult values ranged from 16.6 and 19.8‰. Seasonal trends for δ^34^S values were significant (HGAM; p < 0.05, Supplemental Table S9) for both size classes and followed similar patterns. Sub-adult δ^34^S values were relatively low in spring (18.2‰), but increased to a peak in late summer/early fall (19.3‰) before declining again in winter. A comparable pattern was observed for adult yellowfin tuna, with δ^34^S values increasing from a low in spring (18.3‰) to a peak in late fall (19.0‰). Thus, adult δ^34^S values peaked 1–2 months later than sub-adults; however, the magnitude of change in δ^34^S values was greater in sub-adults.

### Bayesian stable isotope mixing models

**Figure 4:**
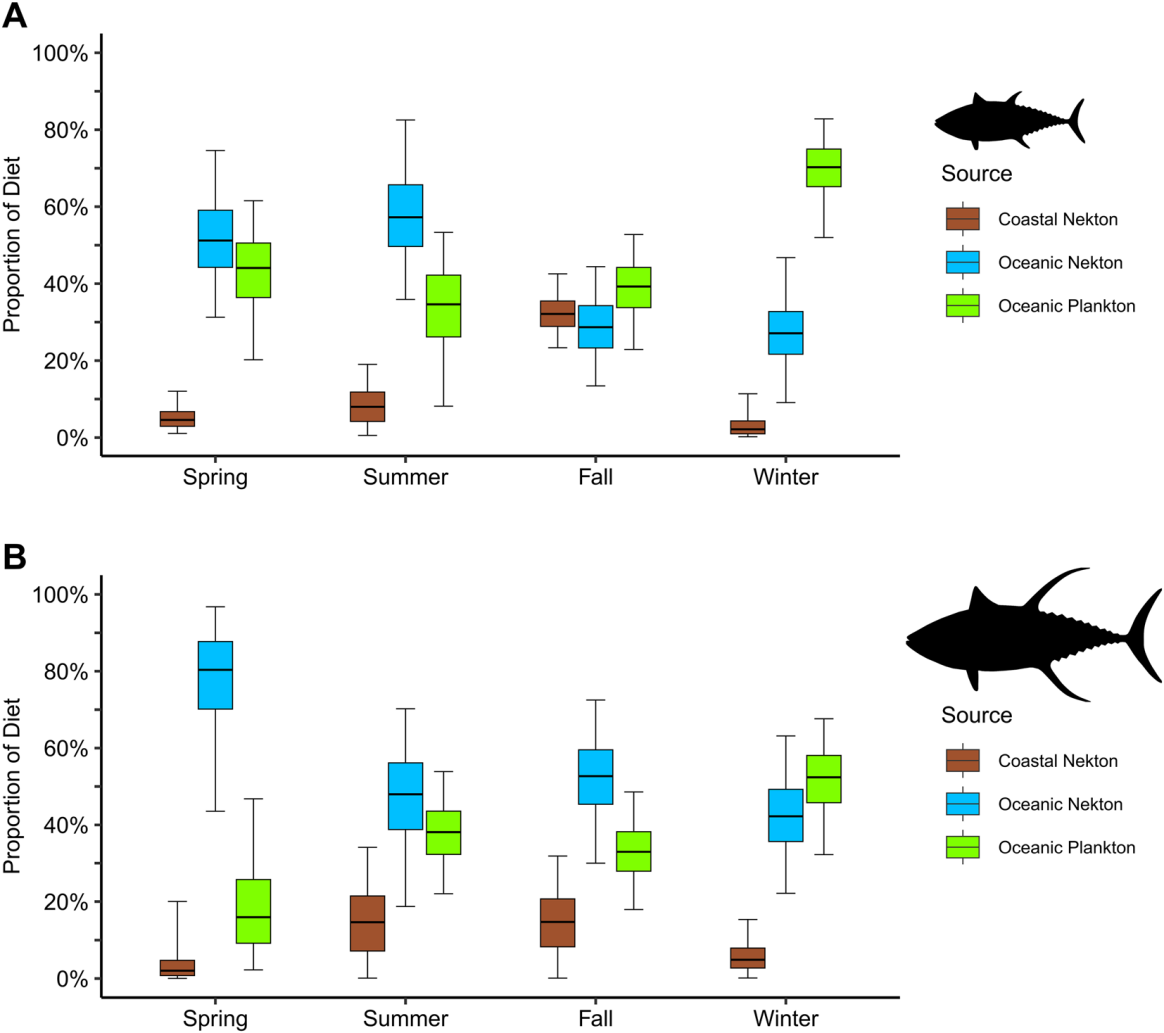
Boxplots (median, interquartile range, and 95% confidence interval error bars) showing the estimated proportional contribution in each season of coastal nekton (brown), oceanic nekton (blue), and oceanic plankton (green) to the diets of sub-adult (**A**) and adult (**B**) yellowfin tuna from the northern Gulf of Mexico (nGoM) based on results of Bayesian stable isotope mixing models (BSIMMs).

BSIMMs were used to estimate the relative contribution (median and 95% confidence interval) of three prey sources to the white muscle tissue of sub-adult and adult yellowfin tuna. Overall, the predicted relative contribution of prey sources to yellowfin tuna diets were seasonally variable between size classes (Fig. 4). Sub-adult diets during spring were influenced by oceanic nekton (51%, CI 31–75%) and oceanic plankton (44%, CI 20–62%), while adult yellowfin tuna diets were overwhelmingly characterized by oceanic nekton (80%, CI 44–97%). During summer, the influence of oceanic nekton (57%, CI 36–83%) increased for sub-adults, but decreased for adult yellowfin tuna (48%, CI 19–70%). The greatest contribution of coastal nekton to sub-adult diets was observed during fall (32%, CI 23–43%), while adults received the greatest coastal nekton contribution during both summer and fall (15%, CI 0–34% and 15%, CI 0–32%, respectively). A noticeable shift occurred during winter as sub-adults received greater contribution from oceanic plankton (70%, CI 52–83%), which was also observed in adult yellowfin tuna diets during winter (42%, CI 22–63%).

## Discussion

### Stomach contents

Yellowfin tuna are opportunistic generalists with a circumtropical distribution, yet previous feeding studies indicate remarkable similarities in diet across multiple ocean basins^41,62–64^. Although yellowfin tuna consume a variety of fishes, squids, and crustaceans, the bulk of the diet in all regions is typically characterized by ommastrephid squids, exocoetids, and scombrids^24,62,65,66^. While these oceanic epipelagic prey were significant components of yellowfin tuna diets in the current study, prey assemblages in the nGoM were notably distinct from other regions^24,41,62–66^ with considerable contribution from planktonic (e.g., hyperiid amphipods, salps, and cavoliniid gastropods), coastal (e.g., mugilids), and reef-associated prey (e.g., serranids, lutjanids, pomacanthids, balistids, and monacanthids). It is possible that these differences simply reflect the high temporal resolution of sampling in the current study (facilitated by a year-round fishery), as the temporal scope of many marine dietary studies is often influenced by external factors (e.g., weather, fishery dynamics, cost, and timing of tournaments or directed scientific sampling) that limit sampling. Another explanation may be the presence of oil and gas platforms, as the aggregating effects of structured habitat on marine biomass is well documented, and the substantial midwater habitat provides settlement structure that is highly accessible to pelagic recruits^67^. Indeed, juveniles of structure-dependent species (e.g., carangids, serranids, lutjanids) that recruit to oil and gas platforms^67,68^ were regularly consumed by yellowfin tuna in the current study, suggesting they may forage more heavily on structure-associated prey than those in other regions that rely on oceanic epipelagic prey^62,66^. In the Central Pacific, the importance of reef fish to yellowfin tuna diets increased near islands or FADs, where juvenile reef fish were more likely to recruit^24,69,70^. It should also be noted that the application of DNA barcoding greatly improved the identification of several juvenile fishes, particularly post-settlement reef fishes (e.g., serranids, lutjanids, pomacanthids, balistids, and monacanthids), which otherwise would have been categorized as unidentified fishes using visual techniques. Still, the importance of structure-associated fishes and planktonic prey (that exhibit positive phototaxis towards artificial lights on platforms)^71^ to nGoM yellowfin tuna suggests regional diet differences could be explained by unique habitat features in the nGoM, such as oil and gas platforms^67,68^, proximity to the Mississippi River Delta, and detached mesoscale features (e.g., eddies) from the Loop Current^21^.

Opportunistic consumption of (small-bodied) planktonic prey has been documented in a range of piscivorous oceanic predators, including several species of tunas^8,72,73^. For example, small crustaceans, such as crab megalopae, stomatopod larvae, and hyperiid amphipods, have previously been observed in the diets of yellowfin tuna^8,10,66,69^. However, the seasonal dominance of planktonic prey in the diets described here far exceeded that of previous studies, suggesting planktonic prey resources play a prominent role in yellowfin tuna diets in the nGoM. While several hyperiid amphipods (e.g., Phrosinidae, Brachyscelidae, Phronimidae, Platyscelidae, and Oxycephalidae) were among the most frequently consumed taxa, the most important planktonic prey resource for yellowfin tuna was *Phrosina semilunata* (recorded in 77% of sub-adult and 27% of adult stomachs). Although *P. semilunata* has been noted in yellowfin tuna diets from several regions^8,65,66,72^, the species’ importance to yellowfin tuna diets in the nGoM could be enhanced by the presence of artificial structures (i.e., oil and gas platforms), as it also has been documented as an important prey resource for other large predators at artificial habitats in the region^74^. The increased importance of planktonic taxa (e.g., *P. semilunata*, salps, cavoliniid gastropods, and other hyperiid amphipods) as food resources for yellowfin tuna during winter may reflect opportunistic foraging behavior, with individuals taking advantage of planktonic prey that are abundant in the water column during periods when other prey resources are relatively scarce. Similar behavior has been described for both Pacific bluefin tuna (*Thunnus orientalis*) and Atlantic bluefin tuna (*Thunnus thynnus*), which fed increasingly on planktonic prey in spawning grounds where food is believed to be infrequent^75^. Likewise, a recent study in the northwest Atlantic with blackfin tuna (*Thunnus atlanticus*) documented a similar diet shift to small planktonic crustaceans during the winter^8^.

The seasonal variability observed in the feeding ecology of yellowfin tuna in the nGoM demonstrates the temporally dynamic foraging patterns of an opportunistic oceanic predator. While seasonal dietary shifts have been described in several terrestrial predators^11–13^, documentation of such patterns in oceanic predators are relatively limited due to complex life histories and sampling limitations (e.g., dependence on recreational/commercial fisheries, seasonal closures, and weather). Temporal shifts in yellowfin tuna diets in the nGoM likely mirrored the availability and abundance of prey^67^, which may largely be influenced by several factors including prey reproductive cycles (e.g., spawning migrations/aggregations and juvenile recruitment) and the association of yellowfin tuna and their prey to structured habitats (i.e., oil and gas platforms)^76^. The influence of prey reproductive cycles on the diet of aquatic, terrestrial, and avian predators is well documented, as predators often preferentially target young and/or weak individuals^77^. The importance of prey reproductive cycles in the foraging ecology of oceanic predators is likely enhanced by several unique reproductive strategies (e.g., aggregation of individuals to spawn, batch spawning, production of hundreds of thousands of offspring, pelagic larvae, and lack of parental care)^78^ of many marine fishes and invertebrates that lead to distinct temporal patterns in prey abundance. Because many oceanic predators (including tunas) are opportunistic foragers, we might expect patterns of relative abundance of prey taxa in stomachs to reflect in situ fluctuations of prey abundance in the surrounding ecosystem^75^. Indeed, yellowfin tuna in the nGoM foraged on juvenile prey such as post-settlement reef fish (i.e., serranids; late spring/early summer), scombrids (early summer), carangids (summer/fall), stomatopod larvae (summer through late fall), and decapod megalopae (fall), which corresponded with the natural progression (from spring to fall) of their settlement patterns^67^. Interestingly, prominent open-water prey, such as exocoetids and ommastrephid squids, were less important to yellowfin tuna diets during summer when juvenile fishes and invertebrates recruit to structured habitat (i.e., *Sargassum* mats, oil and gas platforms)^79^, suggesting yellowfin tuna may opportunistically switch from open-water prey to structure-associated prey to take advantage of the seasonal abundance of this resource. Similarly, the consumption of larger coastal prey in the fall corresponded with the timing of offshore spawning migrations for several inshore taxa (i.e., mugilids and clupeids)^80,81^. As the availability of juvenile fishes declined in late fall/winter^79^, both sub-adult and adult yellowfin tuna shifted to planktonic prey (e.g., hyperiid amphipods, salps, and stomatopod larvae); however, adult yellowfin tuna also foraged on a variety of open-water fishes (e.g., coryphaenids, nomeids, pomatomids, and exocoetids).

Ontogenetic diet shifts are common in marine fishes as rapid changes in body size during development increase their ability to exploit larger prey resources^24^. As a result, size-dependent differences in the feeding ecology of yellowfin tuna might be expected^10^. While a significant dietary shift for yellowfin tuna at 40–50 cm has been linked to a transition from planktonic to piscivorous feeding^24^, individuals in our study were considerably larger and had likely already made this transition. Still, significant diet differences were observed between sub-adult and adult yellowfin tuna, which may be reflective of different habitat associations between the two size classes. Sub-adult diets were characterized by several prey taxa that are strongly associated with structured habitats. Sub-adult yellowfin tuna fed primarily on juvenile fishes (e.g., carangids and reef-associated fishes) and invertebrates (e.g., stomatopod larvae and pelagic crabs) that recruit to platforms and/or sargassum mats during summer and fall before shifting to hyperiid amphipods that are abundant around artificial structures during winter^74^. While adult yellowfin tuna also consumed these structure-associated prey, adult diets were comprised of prey taxa from various habitats, including larger planktonic prey (e.g., salps), open-water fishes (e.g., coryphaenids and scombrids), and coastal fishes (e.g., pomatomids and mugilids). Similar to other oceanic predators in the nGoM, adult yellowfin tuna exploited shrimp fishery discards (largely sciaenids)^82^ and other coastal prey resources during fall when many estuarine fishes (e.g., mugilids and clupeids) make offshore spawning migrations^80,81^. Coastal fishes were less important to sub-adult diets, which could reflect differences in spatial distribution with age or a size bias in the recreational fishery during fall when larger yellowfin tuna are more common near the shelf break (Eddie Burger; Fish Venice Charters, personal communication). Still, our results suggest that yellowfin tuna associated with structured habitat (e.g., fish aggregating devices—FADs, oil and gas platforms) in the nGoM may forage more closely to structure at smaller sizes and expand their foraging range as predation risk decreases with increasing size; a notion supported by previous studies^83^.

### Stable isotopes and Bayesian mixing models

Seasonal cycles in nitrogen and sulfur stable isotope values in yellowfin tuna were similar for both size classes and characterized by a seasonal minimum in δ^15^N values (maximum for δ^34^S values) during late summer for sub-adults and late fall for adults. Assuming a ~ 6- and 9-month turnover rate for sub-adults and adults, respectively^31^, this pattern is in accord with the observed dietary shift to lower trophic prey (e.g., hyperiid amphipods and other planktonic prey) during the previous winter. The fact lower δ^15^N and δ^34^S values were observed in fall rather than summer for adult yellowfin likely reflects slower tissue turnover in larger fish^31^. Alternatively, seasonal shifts in nitrogen and sulfur could reflect seasonal differences in isotopic baselines for these elements^37^, caused by fluctuations in freshwater input from the Mississippi River. River discharge is typically lowest during the winter^84^, thus we might expect nitrogen isotopic baselines to be lower (higher sulfur isotopic baselines) in the winter, thereby more reflective of oceanic water.

The seasonal stability of δ^13^C values observed in adult yellowfin tuna could suggest that sources of primary production (largely phytoplankton) were temporally stable and that adults remained within this region for an extended period of time, which is corroborated by long-term tracking studies that indicate adult yellowfin tuna exhibit high fidelity to the nGoM^1,19,62^. In contrast, lower δ^13^C values observed in sub-adult yellowfin tuna (relative to adults) from late summer through winter suggests that basal carbon sources may be more variable for younger fish in the nGoM. It could also suggest that some individuals from this size class may have recently inhabited a water mass with different primary producer composition and/or dissolved inorganic carbon pools. This could reflect the contribution of recruits from the Atlantic Ocean in the sub-adult size class, as oceanic δ^13^C values for phytoplankton are typically lower in the tropical Atlantic than in the nGoM^85^. This is supported by recent otolith chemistry studies that estimate up to 50% of yellowfin tuna in the nGoM originate from outside the marginal sea^86^.

Seasonal patterns in the predicted relative contribution of coastal nekton, oceanic nekton, and oceanic plankton sources to yellowfin tuna diets were largely supported by stomach content findings. The relatively high estimated contribution of oceanic nekton to sub-adults during spring and summer and to adults during spring, summer, and fall was in agreement with the abundance of carangids, scombrids, exocoetids, and ommastrephid squids in yellowfin stomachs during this period. The contribution of oceanic plankton was more pronounced in sub-adult yellowfin tuna, and the highest contribution for both size classes was observed during winter when greater proportions of hyperiid amphipods are consumed and platform-associated prey fishes are less abundant^79^. Interestingly, the estimated contribution of oceanic plankton to adults during summer and fall exceeded 30%, suggesting that planktonic prey may be more important components to adult diets than stomach contents alone would suggest. While planktonic prey were indeed observed in stomach contents in all seasons, the relative importance to the diet (%IRI) was much lower during the warmer months, particularly for adult yellowfin tuna. This discrepancy may be explained by faster gastric evacuation rates of soft-bodied prey (e.g., salps, hyperiid amphipods) that are much smaller in size relative to that of larger fish prey^87^, as evacuation rates typically decrease with increasing prey size. Furthermore, because metabolic rates of tunas are positively correlated with temperature^88^, digestion rates are likely faster during summer and fall, which could explain why smaller planktonic prey were less abundant in stomach contents during warmer months. While oceanic nekton and oceanic plankton were the most important contributors to yellowfin tuna diets in all seasons, higher relative contribution of coastal nekton to sub-adults (fall) and adults (summer/fall) aligned with aforementioned increases in the availability of coastal fishes duringe fall (due to offshore spawning migrations and trawl bycatch). This seemingly contradicts the lack of coastal prey observed in sub-adult stomachs during fall and could suggest that smaller individuals, foraging on spawning coastal fishes and shrimp bycatch, are underrepresented in the recreational catch due to angler preference for larger yellowfin tuna. An alternative hypothesis may be that the increased influence of coastal nekton in sub-adults during fall and adults during summer/fall reflects the well-documented use of coastal fishes as chum in the recreational fishery, which is primarily comprised of gulf menhaden (*Brevoortia patronus*) and Atlantic bumper (*Chloroscombrus chrysurus*).

There are several limitations which should be considered in dietary studies. Stomach content analysis is commonly used to investigate feeding ecology^73^, but accurate characterization is often limited by the ability to identify digested prey^89^. In the current study, DNA barcoding was applied to aid in this limitation, resulting in a reduction of unidentified prey from 20.9% (%N) to 7.2% (%N) and adds to a growing body of literature that suggests the application of DNA barcoding can greatly improve diet interpretation in stomach content studies^89^. Bayesian stable isotope mixed modeling has become increasingly common in ecological studies; however, the unintended exclusion of prey sources is often a concern and can affect the interpretation of model results. While it is difficult to account for all prey sources consumed by a generalist predator^90^, the current study evaluated and incorporated isotopic data from a broad range of potential prey taxa (12 different families) to minimize this limitation. Finally, the use of fishery-dependent samples could inflate the importance of prey associated with habitats disproportionately targeted by anglers. However, satellite tagging data (Dance & Falterman, unpublished data) suggests that yellowfin tuna move regularly between artificial structures and open-water habitats, and the fact that dietary patterns observed in stomach contents (short-term measure of diet) were largely corroborated by stable isotopes (long-term measure of diet), indicates such biases were likely minimal.

## Conclusions

These findings highlight the seasonal and size-dependent variability that exists in the feeding ecology of a model oceanic predator. Stomach contents of yellowfin tuna in the nGoM were characterized by seasonally distinct prey assemblages that are influenced by prey reproductive cycles, unique habitat features (i.e., oil and gas platforms) and numerous environmental factors (i.e., freshwater discharge and oceanic currents). Seasonal and size-based variability in the utilization of coastal nekton, oceanic nekton, and oceanic plankton sources in the nGoM demonstrates the complex food web dynamics supporting an opportunistic generalist in an open-ocean ecosystem. This study represents a critical step in understanding the temporal and seasonal patterns of prey availability and resource utilization of an oceanic predator. Given the increasing need for accurate dietary information and trophic linkages to improve ecosystem models (e.g., Ecopath with Ecosim), which underpin ecosystem-based management, future research focused on the application of similar high-resolution patterns in predator diets over multiple years is needed to better characterize the complex temporal dynamics supporting oceanic food webs.

### Supplementary Information

Supplementary Information.

## Data Availability

Any data collected and/or used in this article will be available upon request by emailing lovellpublications@gmail.com.

## References

[1] Rooker, J. R. *et al.* Population connectivity of pelagic megafauna in the Cuba-Mexico-United States triangle. *Sci. Rep.* **9**, 1663 (2019). 30733508 10.1038/s41598-018-38144-8 PMC6367330

[2] Frank, K. T., Petrie, B., Choi, J. S. & Leggett, W. C. Trophic cascades in a formerly cod-dominated ecosystem. *Science* **308**, 1621–1623 (2005). 15947186 10.1126/science.1113075

[3] Baum, J. & Worm, B. Cascading top-down effects of changing oceanic predator abundances. *J. Anim. Ecol.* **78**, 699–714 (2009). 19298616 10.1111/j.1365-2656.2009.01531.x

[4] Drymon, J. M. *et al.* Documentation of Atlantic tarpon (*Megalops atlanticus*) space use and move persistence in the northern Gulf of Mexico facilitated by angler advocates. *Conserv. Sci. Pract.* **3**(2), e331 (2020). 10.1111/csp2.331

[5] Heithaus, M. R., Frid, A., Wirsing, A. J. & Worm, B. Predicting ecological consequences of marine top predator declines. *Trends Ecol. Evol.* **23**, 202–210 (2008). 18308421 10.1016/j.tree.2008.01.003

[6] Dale, J. J. *et al.* Global habitat loss of a highly migratory predator, the blue marlin (*Makaira nigricans*). *Divers. Distrib.* **28**, 2020–2034 (2022). 10.1111/ddi.13606

[7] Hays, G. C. *et al.* Key questions in marine megafauna movement ecology. *Trends Ecol. Evol.* **31**, 463–475 (2016). 26979550 10.1016/j.tree.2016.02.015

[8] Poland, S. J., Scharf, F. S. & Staudinger, M. D. Foraging ecology of large pelagic fishes in the US South Atlantic: Structured piscivory shapes trophic niche variation. *Mar. Eco. Prog. Ser.* **631**, 181–199 (2019). 10.3354/meps13126

[9] Schmidt, N. M. *et al.* Response of an arctic predator guild to collapsing lemming cycles. *Proc. R. Soc. Lon. Ser. B* **279**, 4417–4422 (2012). 10.1098/rspb.2012.1490 PMC3479802 22977153

[10] Ménard, F., Labrune, C., Shin, Y.-J., Asine, A.-S. & Bard, F. Opportunistic predation in tuna: A size-based approach. *Mar. Ecol. Prog. Ser.* **323**, 223–231 (2006). 10.3354/meps323223

[11] Davidson, Z. *et al.* Seasonal diet and prey preference of the African lion in a waterhole-driven semi-arid savanna. *PLoS ONE* **8**, e55182 (2013). 23405121 10.1371/journal.pone.0055182 PMC3566210

[12] Latham, A. D. M., Latham, M. C., Knopff, K. H., Hebblewhite, M. & Boutin, S. Wolves, white-tailed deer, and beaver: Implications of seasonal prey switching for woodland caribou declines. *Ecography* **36**, 1276–1290 (2013). 10.1111/j.1600-0587.2013.00035.x

[13] Stenset, N. E. *et al.* Seasonal and annual variation in the diet of brown bears in the boreal forest of southcentral Sweden. *Wildl. Biol.* **22**(3), 107–116 (2016). 10.2981/wlb.00194

[14] Costalago, D., Navarro, J., Álvarez-Calleja, I. & Palomera, I. Ontogenetic and seasonal changes in the feeding habits and trophic levels of two small pelagic fish species. *Mar. Ecol. Prog. Ser.* **460**, 169–181 (2012). 10.3354/meps09751

[15] Block, B. *et al.* Tracking apex marine predator movements in a dynamic ocean. *Nature* **475**, 86–90 (2011). 21697831 10.1038/nature10082

[16] Clarke, A., Griffiths, H. J., Linse, K., Barnes, D. K. A. & Crame, J. A. How well do we know the Antarctic marine fauna? A preliminary study of macroecological and biogeographical patterns in Southern Ocean gastropod and bivalve molluscs. *Divers. Distrib.* **13**, 620–632 (2007). 10.1111/j.1472-4642.2007.00380.x

[17] Acuña-Marrero, D. *et al.* Residency and movement patterns of an apex predatory shark (*Galeocerdo cuvier*) at the Galapagos Marine Reserve. *PLoS ONE* **12**(8), e0183669 (2017). 28829820 10.1371/journal.pone.0183669 PMC5567640

[18] Schaefer, K. M., Fuller, D. W. & Block, B. A. Movements, behavior, and habitat utilization of yellowfin tuna (*Thunnus**albacares*) in the Pacific Ocean off Baja California, Mexico, determined from archival tag data analyses, including unscented Kalman filtering. *Fish Res.* **112**, 22–37 (2011). 10.1016/j.fishres.2011.08.006

[19] Hoolihan, J. P. *et al.* Vertical and Horizontal Movements of Yellowfin Tuna in the Gulf of Mexico. *Mar. Coast. Fish.* **6**(1), 211–222 (2014). 10.1080/19425120.2014.935900

[20] Schaefer, K. M., Fuller, D. W. & Aldana, G. Movements, behavior, and habitat utilization of yellowfin tuna (*Thunnus albacares*) in waters surrounding the Revillagigedo Islands Archipelago Biosphere Reserve, Mexico. *Fish Oceanogr.* **23**, 65–82 (2014). 10.1111/fog.12047

[21] Hamilton, P., Fargion, G. S. & Biggs, D. C. Loop current eddy paths in the western Gulf of Mexico. *J. Phys. Oceanogr.* **29**, 1180–1207 (1999). 10.1175/1520-0485(1999)029<1180:LCEPIT>2.0.CO;2

[22] Rooker, J. R. *et al.* Spatial, temporal, and habitat related variation in abundance of pelagic fishes in the Gulf of Mexico: Potential implications of the Deepwater Horizon oil spill. *PLoS ONE* **8**(10), e76080 (2013). 24130759 10.1371/journal.pone.0076080 PMC3794940

[23] Cornic, M., Smith, B. L., Kitchens, L. L., Bremer, J. R. A. & Rooker, J. R. Abundance and habitat associations of tuna larvae in the surface water of the Gulf of Mexico. *Hydrobiologia* **806**, 29–46 (2018). 10.1007/s10750-017-3330-0

[24] Le-Alvarado, M. *et al.* Yellowfin tuna (*Thunnus albacares*) foraging habitat and trophic position in the Gulf of Mexico based on intrinsic isotope tracers. *PLoS ONE* **16**(2), e0246082 (2021). 33626056 10.1371/journal.pone.0246082 PMC7904200

[25] Graham, B. S., Grubbs, D., Holland, K. & Popp, B. N. A rapid ontogenetic shift in the diet of juvenile yellowfin tuna from Hawaii. *Mar. Biol.* **150**, 647–658 (2007). 10.1007/s00227-006-0360-y

[26] Pecoraro, C. *et al.* Putting all the pieces together: integrating current knowledge of the biology, ecology, fisheries status, stock structure and management of yellowfin tuna (*Thunnus albacares*). *Rev. Fish Biol. Fish.* **27**, 811–841 (2017). 10.1007/s11160-016-9460-z

[27] Cranswick, D. & Regg, J. Deepwater in the Gulf of Mexico: America's New Frontier. *OCS Report MMS 97–0004.* (U.S. Department of the Interior, Minerals Management. Service, Gulf of Mexico OCS Regional Office, 1997).

[28] Zacharia, P. & Abdurahiman, K. Methods of stomach content analysis of fishes. *Winter School on Towards Ecosystem Based Management of Marine Fisheries—Building Mass Balance Trophic and Simulation Models* **1**, 148–158 (2004).

[29] Ward, R. D., Zemlak, T. S., Innes, B. H., Last, P. R. & Hebert, P. D. N. DNA barcoding Australia’s fish species. *Philos. Trans. R. Soc. Lond. B* **360**, 1847–1857 (2005). 16214743 10.1098/rstb.2005.1716 PMC1609232

[30] Altschul, S. F., Gish, W., Miller, W., Myers, E. W. & Lipman, D. J. Basic local alignment search tool. *J. Mol. Biol.* **215**, 403–410 (1990). 2231712 10.1016/S0022-2836(05)80360-2

[31] Vander Zanden, M. J., Clayton, M. K., Moody, E. K., Solomon, C. T. & Weidel, B. C. Stable isotope turnover and half-life in animal tissues: A literature synthesis. *PLoS ONE* **10**(1), e0116182 (2015). 25635686 10.1371/journal.pone.0116182 PMC4321325

[32] Pacicco, A. E. *et al.* Age and growth of Yellowfin Tuna in the US Gulf of Mexico and western Atlantic. *Mar. Coast. Fish.* **13**, 345–361 (2021). 10.1002/mcf2.10158

[33] Pacicco, A. E. *et al.* Reproductive biology of yellowfin tuna (*Thunnus albacares*) in the northcentral US Gulf of Mexico. *Fish. Res.* **261**, 106620 (2023). 10.1016/j.fishres.2023.106620

[34] R Core Team. *R: A Language and Environment for Statistical Computing*. (R Foundation for Statistical Computing, 2020). https://www.R-project.org/.

[35] Oksanen, J. *et al.**Package ‘Vegan’. Community Ecology Package, Version 2*. (2013). https://github.com/vegandevs/vegan.

[36] Arbizu, P. M. pairwiseAdonis: Pairwise multilevel comparison using adonis. *R package version 0.4.* (2020). https://github.com/pmartinezarbizu/pairwiseAdonis.

[37] Lorrain, A. *et al.* Nitrogen isotopic baselines and implications for estimating foraging habitat and trophic position of yellowfin tuna in the Indian and Pacific Oceans. *Deep Sea Res. II* **113**, 188–198 (2015). 10.1016/j.dsr2.2014.02.003

[38] Fry, B. & Chumchal, M. Μ. Sulfur stable isotope indicators of residency in estuarine fish. *Limnol. Oceanogr.* **56**, 1563–1576 (2011). 10.4319/lo.2011.56.5.1563

[39] Szpak, P. & Buckley, M. Sulfur isotopes (δ^34^S) in Arctic marine mammals: Indicators of benthic vs pelagic foraging?. *Mar. Ecol. Prog. Ser.* **653**, 205–216 (2020). 10.3354/meps13493

[40] Varela, J., Larrañaga, A. & Medina, A. Prey-muscle carbon and nitrogen stable-isotope discrimination factors in Atlantic bluefin tuna (*Thunnus thynnus*). *J. Exp. Mar. Biol. Ecol.* **406**, 21–28 (2011). 10.1016/j.jembe.2011.06.010

[41] Ménard, F., Lorrain, A., Potier, M. & Marsac, F. Isotopic evidence of distinct feeding ecologies and movement patterns in two migratory predators (yellowfin tuna and swordfish) of the western Indian Ocean. *Mar. Biol.* **153**, 141–152 (2007). 10.1007/s00227-007-0789-7

[42] Yanagisawa, F. & Sakai, H. Thermal decomposition of barium sulfate-vanadium-pentoxide-silica glass mixtures for preparation of sulfur dioxide in sulfur isotope ratio measurements. *Anal. Chem.* **55**, 985–987 (1983). 10.1021/ac00257a046

[43] Dance, M. A. & Lovell, M. S. lipid correction for carbon stable isotope analysis of yellowfin tuna. *Fishes* **8**(9), 446 (2023). 10.3390/fishes8090446

[44] Post, D. M. *et al.* Getting to the fat of the matter: Models, methods and assumptions for dealing with lipids in stable isotope analyses. *Oecologia* **152**, 179–189 (2007). 17225157 10.1007/s00442-006-0630-x

[45] Logan, J. M. *et al.* Lipid corrections in carbon and nitrogen stable isotope analyses: Comparison of chemical extraction and modelling methods. *J. Anim. Ecol.* **77**, 838–846 (2008). 18489570 10.1111/j.1365-2656.2008.01394.x

[46] Pomerleau, C., Winkler, G., Sastri, A., Nelson, R. J. & Williams, W. J. The effect of acidification and the combined effects of acidification/lipid extraction on carbon stable isotope ratios for sub-arctic and arctic marine zooplankton studies. *Pol. Biol.* **37**, 1541–1548 (2014). 10.1007/s00300-014-1540-8

[47] D’Ambra, I., Graham, W. M., Carmichael, R. H. & Hernandez, F. J. Jr. Dietary overlap between jellyfish and forage fish in the northern Gulf of Mexico. *Mar. Ecol. Prog. Ser.* **587**, 31–40 (2018). 10.3354/meps12419

[48] Pedersen, E. J., Miller, D. L., Simpson, G. L. & Ross, N. Hierarchical generalized additive models in ecology: An introduction with mgcv. *PeerJ.* **7**, e6876 (2019). 31179172 10.7717/peerj.6876 PMC6542350

[49] Wood, S. N. Fast stable restricted maximum likelihood and marginal likelihood estimation of semiparametric generalized linear models. *J. R. Stat. Soc. B* **73**, 3–36 (2011). 10.1111/j.1467-9868.2010.00749.x

[50] Wood, S. N. *Generalized Additive Models: An Introduction with R* 2nd edn. (Taylor and Francis, 2017).

[51] Dance, M. A. & Rooker, J. R. Cross-shelf habitat shifts by red snapper (*Lutjanus campechanus*) in the Gulf of Mexico. *PLoS ONE* **14**(3), e0213506 (2019). 30870449 10.1371/journal.pone.0213506 PMC6417787

[52] Stock, B. C. *et al.* Analyzing mixing systems using a new generation of Bayesian tracer mixing models. *PeerJ* **6**, e5096 (2018). 29942712 10.7717/peerj.5096 PMC6015753

[53] Lerner, J. E. *et al.* Evaluating the use of stable isotope analysis to infer the feeding ecology of a growing US gray seal (*Halichoerus grypus*) population. *PLoS ONE* **13**(2), e192241 (2018). 10.1371/journal.pone.0192241 PMC5821315 29466372

[54] Madigan, D. J. *et al.* Tissue turnover rates and isotopic trophic discrimination factors in the endothermic teleost, Pacific Bluefin Tuna (*Thunnus orientalis*). *PLoS ONE* **7**(11), e49220 (2012). 23145128 10.1371/journal.pone.0049220 PMC3492276

[55] Graham, B. S. Trophic dynamics and movements of tuna in the tropical pacific ocean inferred from stable isotope analyses. *Ph.D. Thesis. University of Hawaii* (2007).

[56] Vehtari, A., Gelman, A. & Gabry, J. Practical Bayesian model evaluation using leave-one-out cross-validation and WAIC. *Stat. Comput.* **27**, 1413–1432 (2017). 10.1007/s11222-016-9696-4

[57] Phillips, D. L. & Koch, P. L. Incorporating concentration dependence in stable isotope mixing models. *Oecologia* **130**, 114–125 (2002). 28547016 10.1007/s004420100786

[58] Gelman, A., Carlin, J. B., Stern, H. S. & Rubin, D. B. *Bayesian Data Analysis* 2nd edn. (Chapman & Hall/CRC, 2004).

[59] Kells, V. & Carpenter, K. *A Field Guide to Coastal Fishes: from Maine to Texas* (The Johns Hopkins University Press, 2011).

[60] Perry, H. & Larsen, K. *A Picture Guide to Shelf Invertebrates from the Northern Gulf of Mexico* (Academic Press, 2004).

[61] Young, C. M., Sewell, M. A. & Rice, M. E. *Atlas of Marine Invertebrate Larvae* (Academic Press, 2002).

[62] Rudershausen, P. J. *et al.* Feeding ecology of blue marlins, dolphinfish, yellowfin tuna, and Wahoos from the North Atlantic Ocean and comparisons with other oceans. *Trans. Am. Fish. Soc.* **139**, 1335–1359 (2010). 10.1577/T09-105.1

[63] Olson, R. *et al.* Chapter four-bioenergetics, trophic ecology, and niche separation of tunas. *Adv. Mar. Biol.* **74**, 199–344 (2016). 27573052 10.1016/bs.amb.2016.06.002

[64] Duffy, L. M. *et al.* Global trophic ecology of yellowfin, bigeye, and albacore tunas: Understanding predation on micronekton communities at ocean-basin scales. *Deep Sea Res. Part II* **140**, 55–73 (2017). 10.1016/j.dsr2.2017.03.003

[65] Zudaire, I. *et al.* Variations in the diet and stable isotope ratios during the ovarian development of female yellowfin tuna (*Thunnus albacares*) in the Western Indian Ocean. *Mar. Biol.* **162**, 2363–2377 (2015). 10.1007/s00227-015-2763-0

[66] da Silva, G. B., Hazin, H. G., Hazin, F. H. V. & Vaske, T. Jr. Diet composition of bigeye tuna (*Thunnus obesus*) and yellowfin tuna (*Thunnus albacares*) caught on aggregated schools in the western equatorial Atlantic Ocean. *J. Appl. Ichthyol.* **35**, 1111–1118 (2019). 10.1111/jai.13949

[67] Hernandez, F. J. *et al.* The across-shelf larval, postlarval, and juvenile fish assemblages collected at offshore oil and gas platforms of the Mississippi River Delta. *Am. Fish. Soc. Symp.* **36**, 39–72 (2003).

[68] Fujii, T. Potential influence of offshore oil and gas platforms on the feeding ecology of fish assemblages in the North Sea. *Mar. Eco. Prog. Ser.* **542**, 167–186 (2016). 10.3354/meps11534

[69] Bertrand, A., Bard, F. X. & Josse, E. Tuna food habits related to the micronekton distribution in French Polynesia. *Mar. Biol.* **140**, 1023–1037 (2002). 10.1007/s00227-001-0776-3

[70] Allain, V. *et al.* Interaction between coastal and oceanic ecosystems of the Western and central Pacific Ocean through predator prey relationship studies. *PLoS ONE* **7**(5), e36701 (2012). 22615796 10.1371/journal.pone.0036701 PMC3352925

[71] Keenan, S. F. The importance of zooplankton in the diets of blue runner (*Caranx crysos*) near offshore petroleum platforms in the northern Gulf of Mexico. M*.S. Thesis, Louisiana State University* (2002).

[72] Potier, M. *et al.* Forage fauna in the diet of three large pelagic fishes (lancetfish, swordfish and yellowfin tuna) in the western equatorial Indian Ocean. *Fish. Res.* **83**, 60–72 (2007). 10.1016/j.fishres.2006.08.020

[73] Weng, J. S. *et al.* Feeding ecology of juvenile yellowfin tuna from waters southwest of Taiwan inferred from stomach contents and stable isotope analysis. *Mar. Coast. Fish.* **7**, 537–548 (2015). 10.1080/19425120.2015.1094157

[74] Tarnecki, J. H. & Patterson, W. F. Changes in Red Snapper diet and trophic ecology following the Deepwater Horizon oil spill. *Mar. Coast. Fish.* **7**, 135–147 (2015). 10.1080/19425120.2015.1020402

[75] Shimose, T. & Wells, R. J. D. Feeding Ecology of Bluefin Tunas. In *Biology and Ecology of Bluefin Tuna* (eds Kitagawa, T. & Kimura, S.) 78–97 (CRC Press, 2015).

[76] Price, M. E., Randall, M. T., Sulak, K. J., Edwards, R. E. & Lamont, M. M. Temporal and spatial relationships of yellowfin tuna to deepwater petroleum platforms in the Northern Gulf of Mexico. *Mar. Coast. Fish.* **14**, e10213 (2022). 10.1002/mcf2.10213

[77] Genovart, M. *et al.* The young, the weak and the sick: Evidence of natural selection by predation. *PLoS ONE* **5**, e9774 (2010). 20333305 10.1371/journal.pone.0009774 PMC2841644

[78] Lowerre-Barbieri, S. K., Ganias, K., Saborido-Rey, F., Murua, H. & Hunter, J. R. Reproductive timing in marine fishes: Variability, temporal scales, and methods. *Mar. Coast. Fish.* **3**, 71–91 (2011). 10.1080/19425120.2011.556932

[79] Stanley, D. R. & Wilson, C. A. Seasonal and spatial variation in the abundance and size distribution of fishes associated with a petroleum platform in the northern Gulf of Mexico. *Can. J. Fish. Aquat. Sci.* **54**, 1166–1176 (1997).

[80] Ditty, J. G. & Shaw, R. F. Spatial and temporal distribution of larval striped mullet (*Mugil cephalus*) and white mullet (*M. curema*, Family: Mugilidae) in the northern Gulf of Mexico, with notes on mountain mullet *Agonostomus monticola*. *Bull. Mar. Sci.* **59**, 271–288 (1996).

[81] Brown-Peterson, N. J., Leaf, R. T., Schueller, A. M. & Andres, M. J. Reproductive dynamics of gulf menhaden (*Brevoortia patronus*) in the northern Gulf of Mexico: Effects on stock assessments. *Fish. Bull.* **115**, 284–299 (2017). 10.7755/FB.115.3.2

[82] Alewijnse, S. R. & Wells, R. J. D. Diet of the Blacktip Shark (*Carcharhinus limbatus*) in the Northwestern Gulf of Mexico. *Gulf Caribb. Res.* **31**, 25–30 (2020). 10.18785/gcr.3101.12

[83] Snodgrass, D. J. G., Orbesen, E. S., Walter, J. F., Hoolihan, J. P. & Brown, C. A. Potential impacts of oil production platforms and their function as fish aggregating devices on the biology of highly migratory fish species. *Rev. Fish Biol. Fish.* **30**, 405–422 (2020). 10.1007/s11160-020-09605-z

[84] Ou, Y. *et al.* A numerical investigation of salinity variations in the Barataria Estuary, Louisiana in connection with the Mississippi River and restoration activities. *Estuar. Coast. Shelf Sci.* **245**, 107021 (2020). 10.1016/j.ecss.2020.107021

[85] Magozzi, S., Yool, A., Zanden, H. B. V., Wunder, M. B. & Trueman, C. N. Using ocean models to predict spatial and temporal variation in marine carbon isotopes. *Ecosphere* **8**, e01763 (2017). 10.1002/ecs2.1763

[86] Rooker, J. R. *et al.* Nursery origin of yellowfin tuna in the western Atlantic Ocean: Significance of Caribbean Sea and trans-Atlantic migrants. *Sci. Rep.* **13**, 16277 (2023). 37770551 10.1038/s41598-023-43163-1 PMC10539535

[87] Olson, R. J. & Boggs, C. H. Apex predation by yellowfin tuna (*Thunnus albacares*): Independent estimates from gastric evacuation and stomach contents, bioenergetics, and cesium concentrations. *Can. J. Fish. Aquat. Sci.* **43**, 1760–1775 (1986). 10.1139/f86-220

[88] Klinger, D. H. *et al.* The effect of temperature on postprandial metabolism of yellowfin tuna (*Thunnus albacares*). *Comp. Biochem. Physiol. A* **195**, 32–38 (2016). 10.1016/j.cbpa.2016.01.005 26794613

[89] Dahl, K. A., Patterson, W. F., Robertson, A. & Ortmann, A. C. DNA barcoding significantly improves resolution of invasive lionfish diet in the Northern Gulf of Mexico. *Biol. Invasions* **19**, 1917–1933 (2017). 10.1007/s10530-017-1407-3

[90] Phillips, D. L. *et al.* Best practices for use of stable isotope mixing models in food web studies. *Can. J. Zool.* **92**, 823–835 (2014). 10.1139/cjz-2014-0127

