# Supplementary material for: Seasonal variability in the feeding ecology of an oceanic predator
Source: Sci Rep. 2024 Jul 29;14:17353. doi: 10.1038/s41598-024-63557-z (PMC11286940; doi:10.1038/s41598-024-63557-z)
Supplement: Supplementary file 1 — Supplementary Information. [file 41598_2024_63557_MOESM1_ESM.docx]

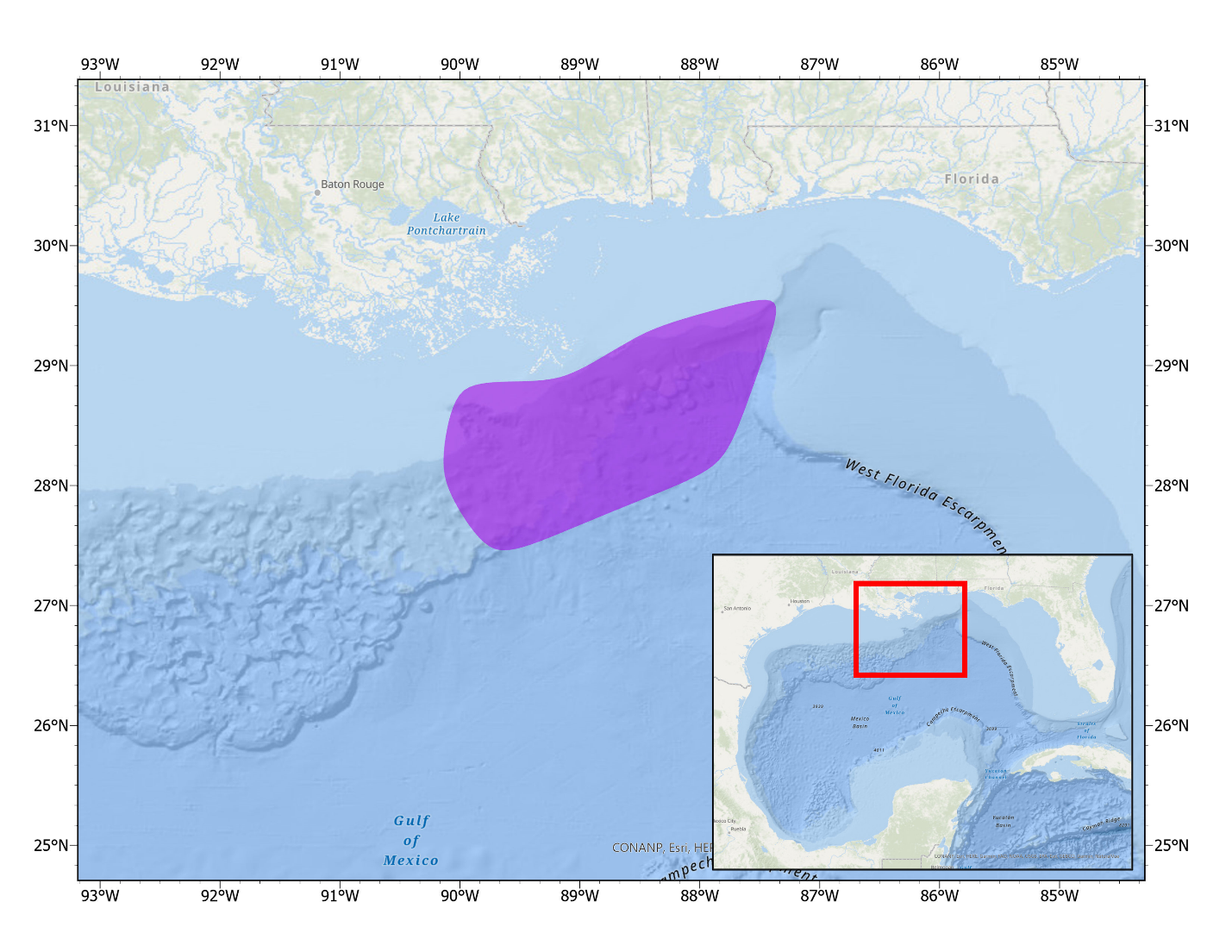


**Supplemental Figure S1.** The estimated area from which yellowfin tuna were captured by recreational charters in the northern Gulf of Mexico (nGoM) from April 2019 to March 2020. The sampling region includes various habitats from both oceanic and coastal ecosystems, of which a large proportion of yellowfin tuna were captured near offshore petroleum infrastructures (e.g., fixed platforms, floating platforms, and drill ships).

**Supplemental Table S1.** The various lipid correction equations used to correct δ^13^C values of the prey taxa incorporated into Bayesian stable isotope mixing models (BSIMMs). The respective source group for each prey taxa and the scientific article from which the equation was referenced is also shown.

| **Source Group** | **Prey Taxa** | **Reference** | **Equation** |  |
| --- | --- | --- | --- | --- |
| Coastal Nekton | Carangidae | Logan et al. 2008 | Eq. 1a: Fish, Muscle - Tissue Specific; Supplemental Material, Appendix S1. |  |
|  | Clupeidae | D'Ambra et al. 2018 | *B. patronus* Equation: (2) |  |
|  | Mugilidae | Logan et al. 2008 | Eq. 1a: Fish, Muscle - Tissue Specific; Supplemental Material, Appendix S1. |  |
| Oceanic Nekton | | Carangidae | Post et al. 2007 | Eq. 3: Aquatic Animals |
|  |  | Exocoetidae | Post et al. 2007 | Eq. 3: Aquatic Animals |
|  |  | Monacanthidae | Post et al. 2007 | Eq. 3: Aquatic Animals |
|  |  | Ommastrephidae | Post et al. 2007 | Eq. 3: Aquatic Animals |
|  |  | Scombridae | Post et al. 2007 | Eq. 3: Aquatic Animals |
| Oceanic Plankton | Brachyscelidae | Pomerleau et al. 2014 | C:N_bulk_ Equation: *Themisto spp.* |  |
|  | Phrosinidae | Pomerleau et al. 2014 | C:N_bulk_ Equation: *Themisto spp.* |  |
|  | Platyscelidae | Pomerleau et al. 2014 | C:N_bulk_ Equation: *Themisto spp.* |  |
|  | Portunidae | Logan et al. 2008 | Eq. 2: Invertebrates, Krill (Euphausiidae); Supplemental Material, Appendix S1. |  |

**Supplemental Table S2.** The mean δ^13^C and δ^15^N values, associated standard deviations (± SD), and associated concentration dependencies (Conc; % carbon and/or % nitrogen) of the prey taxa that were incorporated into Bayesian stable isotope mixing models (BSIMMs, Figure 4) to estimate the relative contribution of coastal nekton, oceanic nekton, and oceanic plankton sources to the diets of sub-adult and adult yellowfin tuna from the northern Gulf of Mexico (nGoM). The sample size (n) for each source group is also shown.

| **Source Group** | **Prey Taxa** | **Mean δ^13^C** | **SD δ^13^C** | **Conc δ^13^C** | **Mean δ^15^N** | **SD δ^15^N** | **Conc δ^15^N** | ***n*** |
| --- | --- | --- | --- | --- | --- | --- | --- | --- |
| Coastal Nekton | Carangidae | -18.2 | 0.438 | 0.475 | 11.2 | 0.519 | 0.134 | *4* |
|  | Clupeidae | -18.5 | 0.566 | 0.482 | 12.8 | 0.589 | 0.121 | *5* |
|  | Mugilidae | -17.3 | 1.876 | 0.461 | 12.3 | 1.866 | 0.142 | *4* |
| Oceanic Nekton | Carangidae | -18.5 | 0.430 | 0.432 | 10.3 | 1.346 | 0.136 | *5* |
|  | Exocoetidae | -18.6 | 0.263 | 0.458 | 11.2 | 1.240 | 0.145 | *4* |
|  | Monacanthidae | -19.0 | 0.550 | 0.471 | 8.4 | 1.197 | 0.149 | *6* |
|  | Ommastrephidae | -18.7 | 0.313 | 0.467 | 10.8 | 1.289 | 0.133 | *6* |
|  | Scombridae | -18.4 | 0.778 | 0.455 | 9.0 | 1.909 | 0.144 | *2* |
| Oceanic Plankton | Brachyscelidae | -17.3 | 1.131 | 0.322 | 7.2 | 0.513 | 0.064 | *3* |
|  | Phrosinidae | -17.6 | 0.675 | 0.312 | 6.9 | 0.591 | 0.077 | *4* |
|  | Platyscelidae | -14.6 | 0.302 | 0.240 | 6.9 | 0.819 | 0.045 | *3* |
|  | Portunidae | -18.4 | 0.409 | 0.376 | 5.6 | 0.356 | 0.055 | *4* |

**Supplemental Table S3.** The mean δ^13^C and δ^15^N values, associated standard deviations (± SD), and associated concentration dependencies (Conc; % carbon and/or % nitrogen) of the three source groups (coastal nekton, oceanic nekton, and oceanic plankton) that were used in Bayesian stable isotope mixing models (BSIMMs, Figure 4) to estimate their relative contribution to the diets of sub-adult and adult yellowfin tuna from the northern Gulf of Mexico (nGoM).

| **Prey Sources** | **Mean δ^13^C** | **SD δ^13^C** | **Conc δ^13^C** | **Mean δ^15^N** | **SD δ^15^N** | **Conc δ^15^N** | ***n*** |
| --- | --- | --- | --- | --- | --- | --- | --- |
| Coastal Nekton | -18.0 | 1.139 | 0.474 | 12.2 | 1.237 | 0.132 | 13 |
| Oceanic Nekton | -18.5 | 0.505 | 0.458 | 10.0 | 1.625 | 0.141 | 23 |
| Oceanic Plankton | -17.7 | 1.762 | 0.317 | 6.6 | 0.835 | 0.061 | 14 |

**Supplemental Table S4.** The seasonal breakdown of sub-adult and adult yellowfin tuna stomachs collected from the northern Gulf of Mexico (nGoM), showing the changes in sample size (n) before and after omitting empty stomachs and stomachs only containing unidentifiable prey (e.g., unidentified fish, squid, crustacean, etc.).

| **Size Class** | **Season** | ***n*** | ***n* after omitting empty stomachs** | ***n* after omitting unidentified prey** |
| --- | --- | --- | --- | --- |
| Sub-adult | Spring | 116 | 113 | 102 |
|  | Summer | 59 | 55 | 34 |
|  | Fall | 93 | 90 | 75 |
|  | Winter | 103 | 102 | 100 |
|  | **Total** | **371** | **360** | **311** |
| Adult | Spring | 30 | 30 | 25 |
|  | Summer | 79 | 79 | 77 |
|  | Fall | 43 | 43 | 39 |
|  | Winter | 54 | 54 | 37 |
|  | **Total** | **206** | **206** | **178** |

**Supplemental Table S5.** The various prey taxa consumed by sub-adult yellowfin tuna in the northern Gulf of Mexico (nGoM), identified to the lowest possible taxonomic grouping (*Genus-species*). Seasonal diet metrics (%N, %FO, %W, and %IRI) for each taxonomic grouping are listed, of which values of prey taxa contributing > 1% to %IRI are bolded. Additionally, broader taxonomic groupings (referred to as Type) are bolded for visualization purposes.

| **Type/Family/*Genus-species*** |  | **Spring** | |  |  |  | **Summer** | |  |  |  | **Fall** | |  |  |  | **Winter** | |  |
| --- | --- | --- | --- | --- | --- | --- | --- | --- | --- | --- | --- | --- | --- | --- | --- | --- | --- | --- | --- |
|  | **%N** | **%FO** | **%W** | **%IRI** |  | **%N** | **%FO** | **%W** | **%IRI** |  | **%N** | **%FO** | **%W** | **%IRI** |  | **%N** | **%FO** | **%W** | **%IRI** |
| **Tunicata** |  |  |  |  |  |  |  |  |  |  |  |  |  |  |  |  |  |  |  |
| Salpidae |  |  |  |  |  |  |  |  |  |  |  |  |  |  |  |  |  |  |  |
| Unidentified Salpidae | 0 | 0 | 0 | 0 |  | 0 | 0 | 0 | 0 |  | 1.90 | 10.67 | 0.10 | 0.52 |  | 6.25 | 38.00 | 0.71 | **3.67** |
| **Gastropoda** |  |  |  |  |  |  |  |  |  |  |  |  |  |  |  |  |  |  |  |
| Cavolinidae |  |  |  |  |  |  |  |  |  |  |  |  |  |  |  |  |  |  |  |
| *Cavolinia tridentata* | 0.32 | 2.94 | 0.03 | 0.02 |  | 0 | 0 | 0 | 0 |  | 1.34 | 10.67 | 0.03 | 0.35 |  | 1.02 | 21.00 | 0.09 | 0.32 |
| *Cavolinia uncinata* | 0.08 | 0.98 | 0.01 | 0.00 |  | 0 | 0 | 0 | 0 |  | 0 | 0 | 0 | 0 |  | 0.08 | 2.00 | 0.01 | 0.00 |
| Vitrinellidae |  |  |  |  |  |  |  |  |  |  |  |  |  |  |  |  |  |  |  |
| Unidentified Vitrinellidae | 0.40 | 3.92 | 0.01 | 0.04 |  | 0 | 0 | 0 | 0 |  | 0.56 | 5.33 | 0.01 | 0.07 |  | 1.74 | 18.00 | 0.11 | 0.46 |
| **Cephalopoda** |  |  |  |  |  |  |  |  |  |  |  |  |  |  |  |  |  |  |  |
| Cranchiidae |  |  |  |  |  |  |  |  |  |  |  |  |  |  |  |  |  |  |  |
| *Cranchia scabra* | 0 | 0 | 0 | 0 |  | 0 | 0 | 0 | 0 |  | 0 | 0 | 0 | 0 |  | 0.51 | 9.00 | 0.13 | 0.08 |
| Enoploteuthidae |  |  |  |  |  |  |  |  |  |  |  |  |  |  |  |  |  |  |  |
| Unidentified Enoploteuthidae | 0 | 0 | 0 | 0 |  | 0 | 0 | 0 | 0 |  | 1.67 | 10.67 | 0.20 | 0.48 |  | 3.51 | 36.00 | 1.60 | **2.56** |
| *Abralia sp.* | 0 | 0 | 0 | 0 |  | 0 | 0 | 0 | 0 |  | 0 | 0 | 0 | 0 |  | 0.03 | 1.00 | 0.14 | 0.00 |
| Octopodidae |  |  |  |  |  |  |  |  |  |  |  |  |  |  |  |  |  |  |  |
| *Macrotritopus defilippi* | 0 | 0 | 0 | 0 |  | 0 | 0 | 0 | 0 |  | 0 | 0 | 0 | 0 |  | 0.54 | 5.00 | 0.16 | 0.05 |
| Ommastrephidae |  |  |  |  |  |  |  |  |  |  |  |  |  |  |  |  |  |  |  |
| Unidentified Ommastrephidae | 2.09 | 10.78 | 1.35 | 0.90 |  | 0 | 0 | 0 | 0 |  | 1.90 | 9.33 | 0.08 | 0.45 |  | 0.35 | 9.00 | 0.57 | 0.11 |
| *Illex sp.* | 1.13 | 4.90 | 2.47 | 0.43 |  | 0 | 0 | 0 | 0 |  | 0.22 | 2.67 | 0.21 | 0.03 |  | 0.16 | 4.00 | 0.23 | 0.02 |
| *Ornithoteuthis antillarum* | 18.36 | 39.22 | 7.15 | **24.27** |  | 9.15 | 23.53 | 0.89 | **2.96** |  | 5.02 | 17.33 | 1.14 | **2.59** |  | 0.94 | 15.00 | 0.95 | 0.39 |
| Sepliolidae |  |  |  |  |  |  |  |  |  |  |  |  |  |  |  |  |  |  |  |
| *Semirossia equalis* | 0 | 0 | 0 | 0 |  | 0 | 0 | 0 | 0 |  | 0 | 0 | 0 | 0 |  | 0.03 | 1.00 | 0.01 | 0.00 |
| **Amphipoda** |  |  |  |  |  |  |  |  |  |  |  |  |  |  |  |  |  |  |  |
| Brachyscelidae |  |  |  |  |  |  |  |  |  |  |  |  |  |  |  |  |  |  |  |
| Unidentified Brachyscelidae | 1.85 | 18.63 | 0.12 | 0.89 |  | 3.92 | 17.65 | 0.07 | 0.88 |  | 14.96 | 34.67 | 0.31 | **12.85** |  | 0.80 | 22.00 | 0.10 | 0.28 |
| Oxycephalidae |  |  |  |  |  |  |  |  |  |  |  |  |  |  |  |  |  |  |  |
| *Streetsia sp.* | 0 | 0 | 0 | 0 |  | 0 | 0 | 0 | 0 |  | 2.57 | 10.67 | 0.07 | 0.68 |  | 0 | 0 | 0 | 0 |
| Phronimidae |  |  |  |  |  |  |  |  |  |  |  |  |  |  |  |  |  |  |  |
| *Phronima sp.* | 2.90 | 19.61 | 0.52 | **1.63** |  | 2.29 | 8.82 | 0.06 | 0.26 |  | 1.90 | 13.33 | 0.18 | 0.67 |  | 0.99 | 18.00 | 0.10 | 0.27 |
| Phrosinidae |  |  |  |  |  |  |  |  |  |  |  |  |  |  |  |  |  |  |  |
| *Phrosina semilunata* | 10.63 | 27.45 | 0.92 | **7.69** |  | 0.33 | 2.94 | 0.02 | 0.01 |  | 4.58 | 30.67 | 0.19 | **3.55** |  | 59.32 | 77.00 | 7.96 | **71.93** |
| Platyscelidae |  |  |  |  |  |  |  |  |  |  |  |  |  |  |  |  |  |  |  |
| *Platyscelus ovoides* | 2.09 | 17.65 | 0.19 | 0.98 |  | 0.33 | 2.94 | 0.00 | 0.01 |  | 2.79 | 26.67 | 0.13 | **1.89** |  | 0.56 | 13.00 | 0.08 | 0.12 |
| **Decapoda** |  |  |  |  |  |  |  |  |  |  |  |  |  |  |  |  |  |  |  |
| Aristeidae |  |  |  |  |  |  |  |  |  |  |  |  |  |  |  |  |  |  |  |
| *Cerataspis monstrosus* | 0.48 | 3.92 | 0.06 | 0.05 |  | 10.46 | 2.94 | 0.38 | 0.40 |  | 0 | 0 | 0 | 0 |  | 0 | 0 | 0 | 0 |
| Ocypodidae |  |  |  |  |  |  |  |  |  |  |  |  |  |  |  |  |  |  |  |
| Unidentified Ocypodidae megalopa | 0 | 0 | 0 | 0 |  | 0 | 0 | 0 | 0 |  | 9.26 | 21.33 | 0.13 | **4.87** |  | 0 | 0 | 0 | 0 |
| Oplophoridae |  |  |  |  |  |  |  |  |  |  |  |  |  |  |  |  |  |  |  |
| *Oplophorus gracilirostris* | 0 | 0 | 0 | 0 |  | 0 | 0 | 0 | 0 |  | 0 | 0 | 0 | 0 |  | 0.03 | 1.00 | 0.08 | 0.00 |
| Paguridae |  |  |  |  |  |  |  |  |  |  |  |  |  |  |  |  |  |  |  |
| Unidentified Paguridae | 0 | 0 | 0 | 0 |  | 0.98 | 5.88 | 0.02 | 0.07 |  | 0.11 | 1.33 | 0.01 | 0.00 |  | 0 | 0 | 0 | 0 |
| Portunidae |  |  |  |  |  |  |  |  |  |  |  |  |  |  |  |  |  |  |  |
| Unidentified Portunidae megalopa | 0 | 0 | 0 | 0 |  | 1.63 | 5.88 | 0.02 | 0.12 |  | 1.90 | 10.67 | 0.03 | 0.50 |  | 1.69 | 13.00 | 0.09 | 0.32 |
| *Callinectes sp.* | 0 | 0 | 0 | 0 |  | 1.31 | 11.76 | 0.35 | 0.24 |  | 0.11 | 1.33 | 0.19 | 0.01 |  | 0 | 0 | 0 | 0 |
| *Portunus gibbesii* | 0.08 | 0.98 | 0.08 | 0.00 |  | 0 | 0 | 0 | 0 |  | 0 | 0 | 0 | 0 |  | 0 | 0 | 0 | 0 |
| *Portunus sayi* | 1.45 | 12.75 | 1.51 | 0.91 |  | 0 | 0 | 0 | 0 |  | 0.33 | 4.00 | 0.11 | 0.04 |  | 0.08 | 3.00 | 0.08 | 0.01 |
| Scyllaridae |  |  |  |  |  |  |  |  |  |  |  |  |  |  |  |  |  |  |  |
| Unidentified Scyllaridae | 0.24 | 2.94 | 0.15 | 0.03 |  | 0 | 0 | 0 | 0 |  | 0.11 | 1.33 | 0.01 | 0.00 |  | 0.05 | 2.00 | 0.02 | 0.00 |
| Xanthidae |  |  |  |  |  |  |  |  |  |  |  |  |  |  |  |  |  |  |  |
| Unidentified Xanthidae megalopa | 0.56 | 3.92 | 0.04 | 0.06 |  | 1.31 | 5.88 | 0.01 | 0.10 |  | 2.79 | 13.33 | 0.04 | 0.92 |  | 0 | 0 | 0 | 0 |
| **Stomatopoda** |  |  |  |  |  |  |  |  |  |  |  |  |  |  |  |  |  |  |  |
| Unidentified Stomatopoda larvae | 0 | 0 | 0 | 0 |  | 22.88 | 41.18 | 0.35 | **11.97** |  | 15.29 | 29.33 | 0.52 | **11.27** |  | 1.31 | 22.00 | 0.22 | 0.47 |
| **Teleostei** |  |  |  |  |  |  |  |  |  |  |  |  |  |  |  |  |  |  |  |
| Ariommatidae |  |  |  |  |  |  |  |  |  |  |  |  |  |  |  |  |  |  |  |
| *Ariomma bondi* | 2.66 | 8.82 | 0.31 | 0.64 |  | 0.65 | 2.94 | 0.03 | 0.03 |  | 1.79 | 2.67 | 0.08 | 0.12 |  | 1.50 | 13.00 | 0.27 | 0.32 |
| Balistidae |  |  |  |  |  |  |  |  |  |  |  |  |  |  |  |  |  |  |  |
| *Balistes capriscus* | 0.24 | 1.96 | 0.09 | 0.02 |  | 0.33 | 2.94 | 0.67 | 0.04 |  | 0 | 0 | 0 | 0 |  | 0 | 0 | 0 | 0 |
| *Xanthichthys ringens* | 0.24 | 1.96 | 0.31 | 0.03 |  | 0 | 0 | 0 | 0 |  | 0.11 | 1.33 | 0.04 | 0.01 |  | 0 | 0 | 0 | 0 |
| Belonidae |  |  |  |  |  |  |  |  |  |  |  |  |  |  |  |  |  |  |  |
| Unidentified Belonidae | 0.16 | 1.96 | 0.70 | 0.04 |  | 0 | 0 | 0 | 0 |  | 0 | 0 | 0 | 0 |  | 0 | 0 | 0 | 0 |
| Caproidae |  |  |  |  |  |  |  |  |  |  |  |  |  |  |  |  |  |  |  |
| *Antigonia capros* | 0.08 | 0.98 | 0.01 | 0.00 |  | 0.33 | 2.94 | 0.06 | 0.01 |  | 0.45 | 4.00 | 0.10 | 0.05 |  | 0 | 0 | 0 | 0 |
| Carangidae |  |  |  |  |  |  |  |  |  |  |  |  |  |  |  |  |  |  |  |
| Unidentified Carangidae | 4.67 | 5.88 | 7.07 | **1.67** |  | 0.33 | 2.94 | 0.02 | 0.01 |  | 0.45 | 4.00 | 0.48 | 0.09 |  | 3.35 | 14.00 | 5.73 | **1.77** |
| *Caranx crysos* | 0.08 | 0.98 | 0.55 | 0.01 |  | 28.43 | 58.82 | 78.24 | **78.56** |  | 2.01 | 13.33 | 16.00 | **5.83** |  | 0.03 | 1.00 | 0.30 | 0.00 |
| *Carangoides ruber* | 0.16 | 0.98 | 0.21 | 0.01 |  | 0 | 0 | 0 | 0 |  | 0 | 0 | 0 | 0 |  | 0 | 0 | 0 | 0 |
| *Decapterus sp.* | 0 | 0 | 0 | 0 |  | 0 | 0 | 0 | 0 |  | 0.89 | 1.33 | 1.05 | 0.06 |  | 0.13 | 1.00 | 0.61 | 0.01 |
| *Decapterus punctatus* | 0 | 0 | 0 | 0 |  | 0 | 0 | 0 | 0 |  | 0.22 | 2.67 | 0.87 | 0.07 |  | 0 | 0 | 0 | 0 |
| *Decapterus tabl* | 0 | 0 | 0 | 0 |  | 0 | 0 | 0 | 0 |  | 0 | 0 | 0 | 0 |  | 2.63 | 6.00 | 2.75 | 0.45 |
| *Hemicaranx amblyrhynchus* | 0 | 0 | 0 | 0 |  | 0 | 0 | 0 | 0 |  | 0.56 | 4.00 | 1.39 | 0.19 |  | 0 | 0 | 0 | 0 |
| *Selar crumenophthalmus* | 0 | 0 | 0 | 0 |  | 0 | 0 | 0 | 0 |  | 1.12 | 4.00 | 1.54 | 0.26 |  | 0 | 0 | 0 | 0 |
| *Selene setapinnis* | 0 | 0 | 0 | 0 |  | 6.54 | 26.47 | 2.15 | **2.88** |  | 8.26 | 26.67 | 13.40 | **14.03** |  | 0 | 0 | 0 | 0 |
| *Seriola dumerili* | 0.08 | 0.98 | 4.15 | 0.10 |  | 0 | 0 | 0 | 0 |  | 0 | 0 | 0 | 0 |  | 0 | 0 | 0 | 0 |
| *Seriola rivoliana* | 0.08 | 0.98 | 1.97 | 0.05 |  | 0 | 0 | 0 | 0 |  | 0 | 0 | 0 | 0 |  | 0 | 0 | 0 | 0 |
| *Trachurus lathami* | 0 | 0 | 0 | 0 |  | 0 | 0 | 0 | 0 |  | 0 | 0 | 0 | 0 |  | 2.28 | 5.00 | 4.67 | 0.48 |
| Clupeidae |  |  |  |  |  |  |  |  |  |  |  |  |  |  |  |  |  |  |  |
| *Brevoortia patronus* | 0 | 0 | 0 | 0 |  | 0 | 0 | 0 | 0 |  | 0.45 | 1.33 | 13.49 | 0.45 |  | 0 | 0 | 0 | 0 |
| *Sardinella aurita* | 0 | 0 | 0 | 0 |  | 0 | 0 | 0 | 0 |  | 0 | 0 | 0 | 0 |  | 0.27 | 4.00 | 0.79 | 0.06 |
| Coryphaenidae |  |  |  |  |  |  |  |  |  |  |  |  |  |  |  |  |  |  |  |
| *Coryphaena sp.* | 0.97 | 5.88 | 5.80 | 0.97 |  | 0 | 0 | 0 | 0 |  | 0.11 | 1.33 | 0.79 | 0.03 |  | 0.08 | 3.00 | 1.63 | 0.07 |
| Diodontidae |  |  |  |  |  |  |  |  |  |  |  |  |  |  |  |  |  |  |  |
| Unidentified Diodontidae | 0 | 0 | 0 | 0 |  | 0 | 0 | 0 | 0 |  | 0 | 0 | 0 | 0 |  | 0.03 | 1.00 | 0.00 | 0.00 |
| *Diodon eydouxii* | 0.08 | 0.98 | 0.32 | 0.01 |  | 0 | 0 | 0 | 0 |  | 0 | 0 | 0 | 0 |  | 0 | 0 | 0 | 0 |
| Echeneidae |  |  |  |  |  |  |  |  |  |  |  |  |  |  |  |  |  |  |  |
| *Echeneis sp.* | 0 | 0 | 0 | 0 |  | 0 | 0 | 0 | 0 |  | 0.11 | 1.33 | 0.01 | 0.00 |  | 0 | 0 | 0 | 0 |
| Exocoetidae |  |  |  |  |  |  |  |  |  |  |  |  |  |  |  |  |  |  |  |
| Unidentified Exocoetidae | 7.49 | 30.39 | 41.80 | **36.34** |  | 0.98 | 5.88 | 3.36 | 0.32 |  | 6.36 | 28.00 | 45.24 | **35.10** |  | 0.97 | 18.00 | 42.71 | **10.92** |
| Gempylidae |  |  |  |  |  |  |  |  |  |  |  |  |  |  |  |  |  |  |  |
| Unidentified Gempylidae | 0 | 0 | 0 | 0 |  | 0.33 | 2.94 | 0.01 | 0.01 |  | 0.22 | 2.67 | 0.02 | 0.02 |  | 1.13 | 21.00 | 1.17 | 0.67 |
| *Gempylus serpens* | 0.24 | 1.96 | 0.11 | 0.02 |  | 0 | 0 | 0 | 0 |  | 0 | 0 | 0 | 0 |  | 0.08 | 3.00 | 0.07 | 0.01 |
| *Promethichthy prometheus* | 0 | 0 | 0 | 0 |  | 0 | 0 | 0 | 0 |  | 0 | 0 | 0 | 0 |  | 0.03 | 1.00 | 0.04 | 0.00 |
| Hemiramphidae |  |  |  |  |  |  |  |  |  |  |  |  |  |  |  |  |  |  |  |
| Unidentified Hemiramphidae | 0.08 | 0.98 | 1.44 | 0.04 |  | 0 | 0 | 0 | 0 |  | 0 | 0 | 0 | 0 |  | 0 | 0 | 0 | 0 |
| *Hemiramphus brasiliensis* | 0.16 | 1.96 | 3.43 | 0.17 |  | 0 | 0 | 0 | 0 |  | 0 | 0 | 0 | 0 |  | 0 | 0 | 0 | 0 |
| Holocentridae |  |  |  |  |  |  |  |  |  |  |  |  |  |  |  |  |  |  |  |
| *Holocentrus adscensionis* | 0.08 | 0.98 | 0.08 | 0.00 |  | 0 | 0 | 0 | 0 |  | 0 | 0 | 0 | 0 |  | 0 | 0 | 0 | 0 |
| Lutjanidae |  |  |  |  |  |  |  |  |  |  |  |  |  |  |  |  |  |  |  |
| *Pristipomoides aquilonaris* | 0 | 0 | 0 | 0 |  | 0 | 0 | 0 | 0 |  | 4.80 | 12.00 | 0.50 | **1.55** |  | 0.08 | 2.00 | 0.02 | 0.00 |
| Malacanthidae |  |  |  |  |  |  |  |  |  |  |  |  |  |  |  |  |  |  |  |
| *Malacanthus plumieri* | 1.37 | 8.82 | 0.31 | 0.36 |  | 0 | 0 | 0 | 0 |  | 0.11 | 1.33 | 0.04 | 0.00 |  | 0.11 | 4.00 | 0.07 | 0.01 |
| Monacanthidae |  |  |  |  |  |  |  |  |  |  |  |  |  |  |  |  |  |  |  |
| Unidentified Monacanthidae | 0.16 | 1.96 | 0.41 | 0.03 |  | 0.33 | 2.94 | 0.55 | 0.03 |  | 0.45 | 5.33 | 0.20 | 0.08 |  | 0.03 | 1.00 | 0.01 | 0.00 |
| *Aluterus sp.* | 0 | 0 | 0 | 0 |  | 0 | 0 | 0 | 0 |  | 0.11 | 1.33 | 0.91 | 0.03 |  | 0.03 | 1.00 | 1.13 | 0.02 |
| *Stephanolepis hispidus* | 0.08 | 0.98 | 0.98 | 0.03 |  | 0 | 0 | 0 | 0 |  | 0 | 0 | 0 | 0 |  | 0 | 0 | 0 | 0 |
| Mugilidae |  |  |  |  |  |  |  |  |  |  |  |  |  |  |  |  |  |  |  |
| *Mugil cephalus* | 0.56 | 3.92 | 3.09 | 0.35 |  | 0 | 0 | 0 | 0 |  | 0 | 0 | 0 | 0 |  | 0.08 | 3.00 | 13.91 | 0.58 |
| Myctophidae |  |  |  |  |  |  |  |  |  |  |  |  |  |  |  |  |  |  |  |
| *Lampanectus sp.* | 0 | 0 | 0 | 0 |  | 0 | 0 | 0 | 0 |  | 0 | 0 | 0 | 0 |  | 0.05 | 1.00 | 0.01 | 0.00 |
| Nomeidae |  |  |  |  |  |  |  |  |  |  |  |  |  |  |  |  |  |  |  |
| Unidentified Nomeidae | 1.53 | 7.84 | 0.37 | 0.36 |  | 0 | 0 | 0 | 0 |  | 0.33 | 4.00 | 0.07 | 0.04 |  | 5.23 | 23.00 | 2.02 | **2.31** |
| *Nomeus gronovii* | 0.08 | 0.98 | 0.56 | 0.02 |  | 0 | 0 | 0 | 0 |  | 0 | 0 | 0 | 0 |  | 0.62 | 9.00 | 7.94 | **1.07** |
| *Psenes cyanophrys* | 2.17 | 5.88 | 0.84 | 0.43 |  | 0 | 0 | 0 | 0 |  | 0 | 0 | 0 | 0 |  | 0.13 | 3.00 | 0.20 | 0.01 |
| Pomacanthidae |  |  |  |  |  |  |  |  |  |  |  |  |  |  |  |  |  |  |  |
| Unidentified Pomacanthidae | 0.64 | 4.90 | 0.08 | 0.09 |  | 0.33 | 2.94 | 0.01 | 0.01 |  | 0.56 | 4.00 | 0.04 | 0.06 |  | 0.56 | 9.00 | 0.05 | 0.08 |
| *Holacanthus tricolor* | 0.08 | 0.98 | 0.01 | 0.00 |  | 0 | 0 | 0 | 0 |  | 0 | 0 | 0 | 0 |  | 0 | 0 | 0 | 0 |
| Scombridae |  |  |  |  |  |  |  |  |  |  |  |  |  |  |  |  |  |  |  |
| Unidentified Scombridae | 0 | 0 | 0 | 0 |  | 0 | 0 | 0 | 0 |  | 0 | 0 | 0 | 0 |  | 0.03 | 1.00 | 0.30 | 0.00 |
| *Auxis thazard* | 0 | 0 | 0 | 0 |  | 1.96 | 5.88 | 3.05 | 0.37 |  | 0 | 0 | 0 | 0 |  | 0 | 0 | 0 | 0 |
| *Auxis rochei* | 2.25 | 5.88 | 6.49 | **1.25** |  | 0 | 0 | 0 | 0 |  | 0 | 0 | 0 | 0 |  | 0 | 0 | 0 | 0 |
| *Thunnus atlanticus* | 0 | 0 | 0 | 0 |  | 0.33 | 2.94 | 9.44 | 0.36 |  | 0 | 0 | 0 | 0 |  | 0 | 0 | 0 | 0 |
| Serranidae |  |  |  |  |  |  |  |  |  |  |  |  |  |  |  |  |  |  |  |
| *Baldwinella vivanus* | 29.55 | 24.51 | 2.30 | **18.94** |  | 4.25 | 5.88 | 0.17 | 0.33 |  | 0 | 0 | 0 | 0 |  | 0.05 | 1.00 | 0.02 | 0.00 |
| Tetradontidae |  |  |  |  |  |  |  |  |  |  |  |  |  |  |  |  |  |  |  |
| Unidentified Tetraodontidae | 0.40 | 2.94 | 0.39 | 0.06 |  | 0 | 0 | 0 | 0 |  | 0 | 0 | 0 | 0 |  | 0.05 | 2.00 | 0.03 | 0.00 |
| *Lagocephalus lagocephalus* | 0.16 | 1.96 | 1.05 | 0.06 |  | 0.33 | 2.94 | 0.05 | 0.01 |  | 0 | 0 | 0 | 0 |  | 0 | 0 | 0 | 0 |
| Syngnathidae |  |  |  |  |  |  |  |  |  |  |  |  |  |  |  |  |  |  |  |
| Unidentified Syngnathidae | 0 | 0 | 0 | 0 |  | 0 | 0 | 0 | 0 |  | 0.11 | 1.33 | 0.00 | 0.00 |  | 0 | 0 | 0 | 0 |
| *Hippocampus erectus* | 0.56 | 0.98 | 0.08 | 0.02 |  | 0 | 0 | 0 | 0 |  | 0 | 0 | 0 | 0 |  | 0 | 0 | 0 | 0 |
| Triglidae |  |  |  |  |  |  |  |  |  |  |  |  |  |  |  |  |  |  |  |
| Unidentified Triglidae | 0.16 | 1.96 | 0.07 | 0.01 |  | 0 | 0 | 0 | 0 |  | 1.12 | 5.33 | 0.03 | 0.15 |  | 0.46 | 9.00 | 0.10 | 0.07 |

**Supplemental Table S6.** The various prey taxa consumed by adult yellowfin tuna in the northern Gulf of Mexico (nGoM), identified to the lowest possible taxonomic grouping (Genus-species). Seasonal diet metrics (%N, %FO, %W, and %IRI) for each taxonomic grouping are listed, of which values of prey taxa contributing > 1% to %IRI are bolded. Additionally, broader taxonomic groupings (referred to as Type) are bolded for visualization purposes.

| **Type/Family/*Genus-species*** |  | **Spring** | |  |  |  | **Summer** | |  |  |  | **Fall** | |  |  |  | **Winter** | |  |
| --- | --- | --- | --- | --- | --- | --- | --- | --- | --- | --- | --- | --- | --- | --- | --- | --- | --- | --- | --- |
|  | **%N** | **%FO** | **%W** | **%IRI** |  | **%N** | **%FO** | **%W** | **%IRI** |  | **%N** | **%FO** | **%W** | **%IRI** |  | **%N** | **%FO** | **%W** | **%IRI** |
| **Echinodermata** |  |  |  |  |  |  |  |  |  |  |  |  |  |  |  |  |  |  |  |
| Astropectinidae |  |  |  |  |  |  |  |  |  |  |  |  |  |  |  |  |  |  |  |
| *Astropecten articulatus* | 0 | 0 | 0 | 0 |  | 0 | 0 | 0 | 0 |  | 0.26 | 2.56 | 0.12 | 0.03 |  | 0 | 0 | 0 | 0 |
| **Tunicata** |  |  |  |  |  |  |  |  |  |  |  |  |  |  |  |  |  |  |  |
| Salpidae |  |  |  |  |  |  |  |  |  |  |  |  |  |  |  |  |  |  |  |
| Unidentified Salpidae | 0 | 0 | 0 | 0 |  | 0 | 0 | 0 | 0 |  | 9.18 | 20.51 | 0.03 | **6.02** |  | 13.16 | 27.03 | 0.14 | **12.00** |
| **Gastropoda** |  |  |  |  |  |  |  |  |  |  |  |  |  |  |  |  |  |  |  |
| Cavolinidae |  |  |  |  |  |  |  |  |  |  |  |  |  |  |  |  |  |  |  |
| *Cavolinia tridentata* | 0 | 0 | 0 | 0 |  | 0.09 | 1.30 | 0.00 | 0.00 |  | 0.26 | 2.56 | 0.00 | 0.02 |  | 0.39 | 5.41 | 0.01 | 0.07 |
| Epitoniidae |  |  |  |  |  |  |  |  |  |  |  |  |  |  |  |  |  |  |  |
| *Janthina janthina* | 0 | 0 | 0 | 0 |  | 0 | 0 | 0 | 0 |  | 0 | 0 | 0 | 0 |  | 5.11 | 2.70 | 0.16 | 0.48 |
| Vitrinellidae |  |  |  |  |  |  |  |  |  |  |  |  |  |  |  |  |  |  |  |
| Unidentified Vitrinellidae | 0 | 0 | 0 | 0 |  | 0 | 0 | 0 | 0 |  | 0 | 0 | 0 | 0 |  | 0.20 | 2.70 | 0.00 | 0.02 |
| **Cephalopoda** |  |  |  |  |  |  |  |  |  |  |  |  |  |  |  |  |  |  |  |
| Cranchiidae |  |  |  |  |  |  |  |  |  |  |  |  |  |  |  |  |  |  |  |
| *Cranchia scabra* | 0 | 0 | 0 | 0 |  | 0.46 | 5.19 | 0.02 | 0.05 |  | 0.26 | 2.56 | 0.00 | 0.02 |  | 0 | 0 | 0 | 0 |
| Enoploteuthidae |  |  |  |  |  |  |  |  |  |  |  |  |  |  |  |  |  |  |  |
| Unidentified Enoploteuthidae | 0 | 0 | 0 | 0 |  | 0 | 0 | 0 | 0 |  | 1.79 | 7.69 | 0.02 | 0.44 |  | 6.48 | 21.62 | 0.62 | **5.13** |
| Octopodidae |  |  |  |  |  |  |  |  |  |  |  |  |  |  |  |  |  |  |  |
| *Octopus sp.* | 0 | 0 | 0 | 0 |  | 0.09 | 1.30 | 0.00 | 0.00 |  | 0 | 0 | 0 | 0 |  | 0 | 0 | 0 | 0 |
| Ommastrephidae |  |  |  |  |  |  |  |  |  |  |  |  |  |  |  |  |  |  |  |
| Unidentified Ommastrephidae | 9.45 | 32.00 | 8.02 | **8.99** |  | 1.93 | 10.39 | 0.24 | 0.49 |  | 3.06 | 17.95 | 0.02 | **1.77** |  | 1.18 | 8.11 | 2.13 | 0.90 |
| *Illex sp.* | 4.56 | 20.00 | 11.39 | **5.13** |  | 2.49 | 11.69 | 0.89 | 0.86 |  | 1.53 | 2.56 | 0.12 | 0.14 |  | 1.38 | 2.70 | 0.14 | 0.14 |
| *Ornithoteuthis antillarum* | 12.87 | 44.00 | 5.48 | **12.98** |  | 6.08 | 23.38 | 0.57 | **3.40** |  | 7.14 | 17.95 | 0.07 | **4.13** |  | 1.38 | 8.11 | 0.09 | 0.40 |
| **Amphipoda** |  |  |  |  |  |  |  |  |  |  |  |  |  |  |  |  |  |  |  |
| Brachyscelidae |  |  |  |  |  |  |  |  |  |  |  |  |  |  |  |  |  |  |  |
| Unidentified Brachyscelidae | 0.49 | 12.00 | 0.04 | 0.10 |  | 0.09 | 1.30 | 0.00 | 0.00 |  | 0.77 | 7.69 | 0.00 | 0.19 |  | 1.38 | 10.81 | 0.02 | 0.50 |
| Phronimidae |  |  |  |  |  |  |  |  |  |  |  |  |  |  |  |  |  |  |  |
| *Phronima sp.* | 4.40 | 24.00 | 1.17 | **2.15** |  | 0.37 | 5.19 | 0.01 | 0.04 |  | 0.26 | 2.56 | 0.00 | 0.02 |  | 0.20 | 2.70 | 0.01 | 0.02 |
| Phrosinidae |  |  |  |  |  |  |  |  |  |  |  |  |  |  |  |  |  |  |  |
| *Phrosina semilunata* | 2.12 | 28.00 | 0.21 | **1.05** |  | 0.18 | 2.60 | 0.00 | 0.01 |  | 0.26 | 2.56 | 0.00 | 0.02 |  | 22.99 | 27.03 | 0.27 | **20.98** |
| Platyscelidae |  |  |  |  |  |  |  |  |  |  |  |  |  |  |  |  |  |  |  |
| *Platyscelus ovoides* | 0.33 | 8.00 | 0.02 | 0.05 |  | 0.09 | 1.30 | 0.00 | 0.00 |  | 0.77 | 7.69 | 0.00 | 0.19 |  | 0.20 | 2.70 | 0.00 | 0.02 |
| **Decapoda** |  |  |  |  |  |  |  |  |  |  |  |  |  |  |  |  |  |  |  |
| Aristeidae |  |  |  |  |  |  |  |  |  |  |  |  |  |  |  |  |  |  |  |
| *Cerataspis monstrosus* | 0.98 | 12.00 | 0.14 | 0.22 |  | 0.37 | 3.90 | 0.01 | 0.03 |  | 0 | 0 | 0 | 0 |  | 0 | 0 | 0 | 0 |
| Ocypodidae |  |  |  |  |  |  |  |  |  |  |  |  |  |  |  |  |  |  |  |
| Unidentified Ocypodidae megalopa | 0 | 0 | 0 | 0 |  | 1.47 | 3.90 | 0.01 | 0.13 |  | 1.79 | 2.56 | 0.00 | 0.15 |  | 0 | 0 | 0 | 0 |
| Oplophoridae |  |  |  |  |  |  |  |  |  |  |  |  |  |  |  |  |  |  |  |
| *Janicella spinicauda* | 0 | 0 | 0 | 0 |  | 0.09 | 1.30 | 0.00 | 0.00 |  | 0 | 0 | 0 | 0 |  | 0 | 0 | 0 | 0 |
| Penaeidae |  |  |  |  |  |  |  |  |  |  |  |  |  |  |  |  |  |  |  |
| Unidentified Penaeidae | 0 | 0 | 0 | 0 |  | 0 | 0 | 0 | 0 |  | 0.26 | 2.56 | 0.10 | 0.03 |  | 0 | 0 | 0 | 0 |
| *Farfantepenaeus subtilis* | 0 | 0 | 0 | 0 |  | 0 | 0 | 0 | 0 |  | 0 | 0 | 0 | 0 |  | 0.98 | 2.70 | 0.54 | 0.14 |
| Portunidae |  |  |  |  |  |  |  |  |  |  |  |  |  |  |  |  |  |  |  |
| Unidentified Portunidae megalopa | 0 | 0 | 0 | 0 |  | 0.18 | 2.60 | 0.00 | 0.01 |  | 0 | 0 | 0 | 0 |  | 0.20 | 2.70 | 0.01 | 0.02 |
| *Callinectes sp.* | 0 | 0 | 0 | 0 |  | 1.38 | 7.79 | 0.07 | 0.25 |  | 0 | 0 | 0 | 0 |  | 0 | 0 | 0 | 0 |
| *Callinectes sapidus* | 0 | 0 | 0 | 0 |  | 0.18 | 1.30 | 0.20 | 0.01 |  | 1.53 | 5.13 | 0.62 | 0.35 |  | 0 | 0 | 0 | 0 |
| *Portunus sayi* | 1.14 | 16.00 | 1.33 | 0.63 |  | 0.18 | 2.60 | 0.00 | 0.01 |  | 0 | 0 | 0 | 0 |  | 0 | 0 | 0 | 0 |
| Scyllaridae |  |  |  |  |  |  |  |  |  |  |  |  |  |  |  |  |  |  |  |
| Unidentified Scyllaridae | 0.49 | 4.00 | 0.25 | 0.05 |  | 0 | 0 | 0 | 0 |  | 0 | 0 | 0 | 0 |  | 0.20 | 2.70 | 0.00 | 0.02 |
| Xanthidae |  |  |  |  |  |  |  |  |  |  |  |  |  |  |  |  |  |  |  |
| Unidentified Xanthidae megalopa | 0.33 | 8.00 | 0.04 | 0.05 |  | 1.57 | 10.39 | 0.01 | 0.36 |  | 0 | 0 | 0 | 0 |  | 0 | 0 | 0 | 0 |
| **Stomatopoda** |  |  |  |  |  |  |  |  |  |  |  |  |  |  |  |  |  |  |  |
| Unidentified Stomatopoda larvae | 0 | 0 | 0 | 0 |  | 3.04 | 12.99 | 0.05 | 0.88 |  | 0.77 | 5.13 | 0.00 | 0.13 |  | 2.16 | 10.81 | 0.04 | 0.79 |
| Squillidae |  |  |  |  |  |  |  |  |  |  |  |  |  |  |  |  |  |  |  |
| *Squilla empusa* | 0 | 0 | 0 | 0 |  | 0.09 | 1.30 | 0.10 | 0.01 |  | 0 | 0 | 0 | 0 |  | 0 | 0 | 0 | 0 |
| **Teleostei** |  |  |  |  |  |  |  |  |  |  |  |  |  |  |  |  |  |  |  |
| Ariommatidae |  |  |  |  |  |  |  |  |  |  |  |  |  |  |  |  |  |  |  |
| *Ariomma bondi* | 1.14 | 8.00 | 0.16 | 0.17 |  | 1.20 | 5.19 | 0.02 | 0.14 |  | 0 | 0 | 0 | 0 |  | 6.68 | 2.70 | 0.14 | 0.62 |
| Balistidae |  |  |  |  |  |  |  |  |  |  |  |  |  |  |  |  |  |  |  |
| Unidentified Balistidae | 0 | 0 | 0 | 0 |  | 0.09 | 1.30 | 0.12 | 0.01 |  | 0 | 0 | 0 | 0 |  | 0 | 0 | 0 | 0 |
| *Balistes capriscus* | 0.33 | 4.00 | 0.05 | 0.02 |  | 0.18 | 2.60 | 0.26 | 0.03 |  | 0 | 0 | 0 | 0 |  | 0 | 0 | 0 | 0 |
| *Xanthichthys ringens* | 0.33 | 4.00 | 0.10 | 0.03 |  | 0.09 | 1.30 | 0.04 | 0.00 |  | 0.26 | 2.56 | 0.02 | 0.02 |  | 0 | 0 | 0 | 0 |
| Belonidae |  |  |  |  |  |  |  |  |  |  |  |  |  |  |  |  |  |  |  |
| Unidentified Belonidae | 0.81 | 4.00 | 1.36 | 0.14 |  | 0 | 0 | 0 | 0 |  | 0.26 | 2.56 | 0.15 | 0.03 |  | 0 | 0 | 0 | 0 |
| Carangidae |  |  |  |  |  |  |  |  |  |  |  |  |  |  |  |  |  |  |  |
| Unidentified Carangidae | 7.49 | 12.00 | 3.30 | **2.08** |  | 0.74 | 5.19 | 1.05 | 0.20 |  | 0.51 | 5.13 | 0.01 | 0.09 |  | 0 | 0 | 0 | 0 |
| *Caranx sp.* | 0 | 0 | 0 | 0 |  | 0.09 | 1.30 | 0.07 | 0.00 |  | 0 | 0 | 0 | 0 |  | 0 | 0 | 0 | 0 |
| *Caranx crysos* | 0.49 | 4.00 | 12.64 | 0.84 |  | 23.30 | 54.55 | 26.71 | **59.76** |  | 6.12 | 20.51 | 6.31 | **8.13** |  | 3.93 | 8.11 | 13.48 | **4.71** |
| *Carangoides ruber* | 0 | 0 | 0 | 0 |  | 0.09 | 1.30 | 0.84 | 0.03 |  | 0 | 0 | 0 | 0 |  | 0 | 0 | 0 | 0 |
| *Decapterus sp.* | 0 | 0 | 0 | 0 |  | 0.28 | 1.30 | 0.23 | 0.01 |  | 0 | 0 | 0 | 0 |  | 0 | 0 | 0 | 0 |
| *Decapterus punctatus* | 0 | 0 | 0 | 0 |  | 0 | 0 | 0 | 0 |  | 0.26 | 2.56 | 0.03 | 0.02 |  | 0 | 0 | 0 | 0 |
| *Selar crumenophthalmus* | 0 | 0 | 0 | 0 |  | 0 | 0 | 0 | 0 |  | 0.51 | 2.56 | 0.37 | 0.07 |  | 0 | 0 | 0 | 0 |
| *Selene setapinnis* | 0 | 0 | 0 | 0 |  | 7.73 | 23.38 | 0.77 | **4.36** |  | 3.83 | 12.82 | 0.64 | **1.82** |  | 0 | 0 | 0 | 0 |
| *Trachurus lathami* | 0 | 0 | 0 | 0 |  | 0 | 0 | 0 | 0 |  | 0 | 0 | 0 | 0 |  | 7.47 | 5.41 | 0.92 | **1.51** |
| Clupeidae |  |  |  |  |  |  |  |  |  |  |  |  |  |  |  |  |  |  |  |
| *Brevoortia patronus* | 0 | 0 | 0 | 0 |  | 0 | 0 | 0 | 0 |  | 2.81 | 5.13 | 2.31 | 0.84 |  | 1.96 | 5.41 | 0.43 | 0.43 |
| Coryphaenidae |  |  |  |  |  |  |  |  |  |  |  |  |  |  |  |  |  |  |  |
| *Coryphaena sp.* | 0.16 | 4.00 | 1.11 | 0.08 |  | 0.18 | 1.30 | 0.64 | 0.02 |  | 0 | 0 | 0 | 0 |  | 7.86 | 32.43 | 15.33 | **25.10** |
| Dactylopteridae |  |  |  |  |  |  |  |  |  |  |  |  |  |  |  |  |  |  |  |
| *Dactylopterus volitans* | 0 | 0 | 0 | 0 |  | 0.64 | 6.49 | 0.06 | 0.10 |  | 0 | 0 | 0 | 0 |  | 0 | 0 | 0 | 0 |
| Diodontidae |  |  |  |  |  |  |  |  |  |  |  |  |  |  |  |  |  |  |  |
| *Chilomycterus sp.* | 0 | 0 | 0 | 0 |  | 0 | 0 | 0 | 0 |  | 0 | 0 | 0 | 0 |  | 0.20 | 2.70 | 0.12 | 0.03 |
| Exocoetidae |  |  |  |  |  |  |  |  |  |  |  |  |  |  |  |  |  |  |  |
| Unidentified Exocoetidae | 4.72 | 52.00 | 45.23 | **41.77** |  | 0.28 | 2.60 | 1.57 | 0.11 |  | 10.20 | 23.08 | 7.80 | **13.25** |  | 2.95 | 18.92 | 17.67 | **13.02** |
| Gempylidae |  |  |  |  |  |  |  |  |  |  |  |  |  |  |  |  |  |  |  |
| Unidentified Gempylidae | 0 | 0 | 0 | 0 |  | 0.18 | 2.60 | 0.01 | 0.01 |  | 2.04 | 7.69 | 0.11 | 0.53 |  | 1.18 | 8.11 | 0.23 | 0.38 |
| *Gempylus serpens* | 1.30 | 12.00 | 1.41 | 0.52 |  | 1.20 | 9.09 | 0.31 | 0.30 |  | 0 | 0 | 0 | 0 |  | 0 | 0 | 0 | 0 |
| *Nealotus tripes* | 0.16 | 4.00 | 0.86 | 0.07 |  | 0.09 | 1.30 | 0.01 | 0.00 |  | 0.51 | 2.56 | 0.10 | 0.05 |  | 0 | 0 | 0 | 0 |
| Hemiramphidae |  |  |  |  |  |  |  |  |  |  |  |  |  |  |  |  |  |  |  |
| Unidentified Hemiramphidae | 0 | 0 | 0 | 0 |  | 0.09 | 1.30 | 0.22 | 0.01 |  | 0 | 0 | 0 | 0 |  | 0.20 | 2.70 | 0.70 | 0.08 |
| Holocentridae |  |  |  |  |  |  |  |  |  |  |  |  |  |  |  |  |  |  |  |
| *Holocentrus adscensionis* | 0.98 | 4.00 | 0.26 | 0.08 |  | 0 | 0 | 0 | 0 |  | 0 | 0 | 0 | 0 |  | 0 | 0 | 0 | 0 |
| Lobotidae |  |  |  |  |  |  |  |  |  |  |  |  |  |  |  |  |  |  |  |
| *Lobotes surinamensis* | 0 | 0 | 0 | 0 |  | 0 | 0 | 0 | 0 |  | 0.26 | 2.56 | 4.39 | 0.38 |  | 0 | 0 | 0 | 0 |
| Lutjanidae |  |  |  |  |  |  |  |  |  |  |  |  |  |  |  |  |  |  |  |
| *Pristipomoides aquilonaris* | 0 | 0 | 0 | 0 |  | 0.46 | 2.60 | 0.02 | 0.03 |  | 1.53 | 10.26 | 0.03 | 0.51 |  | 0 | 0 | 0 | 0 |
| Malacanthidae |  |  |  |  |  |  |  |  |  |  |  |  |  |  |  |  |  |  |  |
| *Malacanthus plumieri* | 0.65 | 12.00 | 0.24 | 0.17 |  | 0.09 | 1.30 | 0.01 | 0.00 |  | 0 | 0 | 0 | 0 |  | 0 | 0 | 0 | 0 |
| Monacanthidae |  |  |  |  |  |  |  |  |  |  |  |  |  |  |  |  |  |  |  |
| Unidentified Monacanthidae | 0 | 0 | 0 | 0 |  | 0.09 | 1.30 | 0.08 | 0.00 |  | 1.02 | 7.69 | 0.13 | 0.28 |  | 0.20 | 2.70 | 0.02 | 0.02 |
| *Cantherhines pullus* | 0 | 0 | 0 | 0 |  | 0.09 | 1.30 | 0.10 | 0.01 |  | 0 | 0 | 0 | 0 |  | 0 | 0 | 0 | 0 |
| Mugilidae |  |  |  |  |  |  |  |  |  |  |  |  |  |  |  |  |  |  |  |
| *Mugil cephalus* | 0 | 0 | 0 | 0 |  | 0 | 0 | 0 | 0 |  | 12.76 | 23.08 | 55.99 | **50.60** |  | 0.59 | 2.70 | 19.18 | **1.78** |
| Myctophidae |  |  |  |  |  |  |  |  |  |  |  |  |  |  |  |  |  |  |  |
| *Lampanectus sp.* | 0 | 0 | 0 | 0 |  | 0 | 0 | 0 | 0 |  | 0.26 | 2.56 | 0.02 | 0.02 |  | 0 | 0 | 0 | 0 |
| Nomeidae |  |  |  |  |  |  |  |  |  |  |  |  |  |  |  |  |  |  |  |
| Unidentified Nomeidae | 0.65 | 12.00 | 0.48 | 0.22 |  | 5.89 | 5.19 | 0.39 | 0.72 |  | 6.12 | 10.26 | 0.13 | **2.05** |  | 4.32 | 8.11 | 0.15 | **1.21** |
| *Cubiceps pauciradiatus* | 0 | 0 | 0 | 0 |  | 0 | 0 | 0 | 0 |  | 0 | 0 | 0 | 0 |  | 0.39 | 2.70 | 0.94 | 0.12 |
| *Nomeus gronovii* | 1.79 | 4.00 | 1.83 | 0.23 |  | 0 | 0 | 0 | 0 |  | 0 | 0 | 0 | 0 |  | 0 | 0 | 0 | 0 |
| *Psenes cyanophrys* | 0.16 | 4.00 | 0.05 | 0.01 |  | 0 | 0 | 0 | 0 |  | 0 | 0 | 0 | 0 |  | 0.98 | 8.11 | 0.19 | 0.32 |
| Ostraciidae |  |  |  |  |  |  |  |  |  |  |  |  |  |  |  |  |  |  |  |
| Unidentified Ostraciidae | 0 | 0 | 0 | 0 |  | 0.09 | 1.30 | 0.01 | 0.00 |  | 0 | 0 | 0 | 0 |  | 0 | 0 | 0 | 0 |
| Polymixiidae |  |  |  |  |  |  |  |  |  |  |  |  |  |  |  |  |  |  |  |
| *Polymixia lowei* | 0 | 0 | 0 | 0 |  | 0.09 | 1.30 | 0.04 | 0.00 |  | 0 | 0 | 0 | 0 |  | 0 | 0 | 0 | 0 |
| Pomacanthidae |  |  |  |  |  |  |  |  |  |  |  |  |  |  |  |  |  |  |  |
| Unidentified Pomacanthidae | 0.33 | 8.00 | 0.02 | 0.05 |  | 0.28 | 2.60 | 0.01 | 0.02 |  | 0.77 | 5.13 | 0.01 | 0.13 |  | 0 | 0 | 0 | 0 |
| *Centropyge argi* | 0.33 | 4.00 | 0.01 | 0.02 |  | 0 | 0 | 0 | 0 |  | 0 | 0 | 0 | 0 |  | 0 | 0 | 0 | 0 |
| Pomatomidae |  |  |  |  |  |  |  |  |  |  |  |  |  |  |  |  |  |  |  |
| *Pomatomus saltatrix* | 0 | 0 | 0 | 0 |  | 0 | 0 | 0 | 0 |  | 0 | 0 | 0 | 0 |  | 1.38 | 10.81 | 21.83 | **8.37** |
| Sciaeneidae |  |  |  |  |  |  |  |  |  |  |  |  |  |  |  |  |  |  |  |
| Unidentified Sciaenidae | 0 | 0 | 0 | 0 |  | 0 | 0 | 0 | 0 |  | 0 | 0 | 0 | 0 |  | 0.39 | 2.70 | 1.69 | 0.19 |
| *Cynoscion sp.* | 0 | 0 | 0 | 0 |  | 0.64 | 2.60 | 4.73 | 0.31 |  | 0 | 0 | 0 | 0 |  | 0 | 0 | 0 | 0 |
| *Cynoscion arenarius* | 0 | 0 | 0 | 0 |  | 0 | 0 | 0 | 0 |  | 0.51 | 2.56 | 0.76 | 0.10 |  | 0 | 0 | 0 | 0 |
| *Leiostomus xanthurus* | 0 | 0 | 0 | 0 |  | 0 | 0 | 0 | 0 |  | 0.26 | 2.56 | 0.57 | 0.07 |  | 0 | 0 | 0 | 0 |
| *Micropognias undulatus* | 0 | 0 | 0 | 0 |  | 5.89 | 2.60 | 20.22 | **1.49** |  | 13.27 | 7.69 | 3.06 | **4.00** |  | 0.20 | 2.70 | 0.19 | 0.03 |
| Scombridae |  |  |  |  |  |  |  |  |  |  |  |  |  |  |  |  |  |  |  |
| Unidentified Scombridae | 0 | 0 | 0 | 0 |  | 0.09 | 1.30 | 1.02 | 0.03 |  | 0 | 0 | 0 | 0 |  | 0.20 | 2.70 | 2.47 | 0.24 |
| *Auxis thazard* | 0 | 0 | 0 | 0 |  | 16.85 | 29.87 | 11.13 | **18.31** |  | 0.26 | 2.56 | 0.04 | 0.02 |  | 0 | 0 | 0 | 0 |
| *Auxis sp.* | 0 | 0 | 0 | 0 |  | 0.28 | 1.30 | 0.15 | 0.01 |  | 0 | 0 | 0 | 0 |  | 0 | 0 | 0 | 0 |
| *Euthynnus alletteratus* | 0 | 0 | 0 | 0 |  | 2.95 | 9.09 | 7.06 | **1.99** |  | 0 | 0 | 0 | 0 |  | 0 | 0 | 0 | 0 |
| *Katsuwonus pelamis* | 0 | 0 | 0 | 0 |  | 0.09 | 1.30 | 0.27 | 0.01 |  | 0 | 0 | 0 | 0 |  | 0 | 0 | 0 | 0 |
| *Thunnus sp.* | 0 | 0 | 0 | 0 |  | 0 | 0 | 0 | 0 |  | 0.26 | 2.56 | 1.26 | 0.12 |  | 0 | 0 | 0 | 0 |
| *Thunnus atlanticus* | 0 | 0 | 0 | 0 |  | 0.64 | 9.09 | 17.96 | **3.71** |  | 0.51 | 5.13 | 12.15 | **2.07** |  | 0 | 0 | 0 | 0 |
| Serranidae |  |  |  |  |  |  |  |  |  |  |  |  |  |  |  |  |  |  |  |
| *Baldwinella vivanus* | 40.23 | 32.00 | 2.61 | **22.04** |  | 5.71 | 10.39 | 0.19 | **1.34** |  | 0 | 0 | 0 | 0 |  | 0 | 0 | 0 | 0 |
| Sparidae |  |  |  |  |  |  |  |  |  |  |  |  |  |  |  |  |  |  |  |
| Unidentified Sparidae | 0 | 0 | 0 | 0 |  | 0.09 | 1.30 | 0.00 | 0.00 |  | 0 | 0 | 0 | 0 |  | 0 | 0 | 0 | 0 |
| *Stenotomus caprinus* | 0 | 0 | 0 | 0 |  | 0 | 0 | 0 | 0 |  | 0.26 | 2.56 | 0.23 | 0.04 |  | 0 | 0 | 0 | 0 |
| Stromateidae |  |  |  |  |  |  |  |  |  |  |  |  |  |  |  |  |  |  |  |
| *Peprilus burti* | 0 | 0 | 0 | 0 |  | 0 | 0 | 0 | 0 |  | 0.51 | 5.13 | 0.98 | 0.24 |  | 0 | 0 | 0 | 0 |
| Syngnathidae |  |  |  |  |  |  |  |  |  |  |  |  |  |  |  |  |  |  |  |
| *Hippocampus erectus* | 0 | 0 | 0 | 0 |  | 0.18 | 2.60 | 0.02 | 0.01 |  | 0.26 | 2.56 | 0.00 | 0.02 |  | 0.20 | 2.70 | 0.03 | 0.02 |
| Tetradontidae |  |  |  |  |  |  |  |  |  |  |  |  |  |  |  |  |  |  |  |
| Unidentified Tetraodontidae | 0.49 | 4.00 | 0.10 | 0.04 |  | 0.64 | 7.79 | 0.03 | 0.12 |  | 0.26 | 2.56 | 0.00 | 0.02 |  | 0 | 0 | 0 | 0 |
| *Lagocephalus sp.* | 0 | 0 | 0 | 0 |  | 0.18 | 1.30 | 0.01 | 0.01 |  | 0 | 0 | 0 | 0 |  | 0 | 0 | 0 | 0 |
| *Lagocephalus lagocephalus* | 0.16 | 4.00 | 0.04 | 0.01 |  | 1.01 | 5.19 | 0.12 | 0.13 |  | 0 | 0 | 0 | 0 |  | 0 | 0 | 0 | 0 |
| Trichiuridae |  |  |  |  |  |  |  |  |  |  |  |  |  |  |  |  |  |  |  |
| *Trichiurus lepturus* | 0 | 0 | 0 | 0 |  | 0.64 | 2.60 | 0.93 | 0.09 |  | 3.83 | 5.13 | 1.17 | 0.82 |  | 0.20 | 2.70 | 0.05 | 0.02 |
| Triglidae |  |  |  |  |  |  |  |  |  |  |  |  |  |  |  |  |  |  |  |
| Unidentified Triglidae | 0.16 | 4.00 | 0.04 | 0.01 |  | 0.09 | 1.30 | 0.36 | 0.01 |  | 0 | 0 | 0 | 0 |  | 0.98 | 5.41 | 0.06 | 0.19 |

**Supplemental Table S7.** The similarity percentage (SIMPER) analysis of sub-adult and adult yellowfin tuna from the northern Gulf of Mexico (nGoM). All seasonal diets were compared to one another for sub-adult and adult dissimilarities, while only intra-seasonal comparisons were made for the sub-adult*adult dissimilarity. In combination, these nine most influential prey taxa contributed at least 50% to each compared diet.

|  | **Diets Compared** | **Carangid** | **Coryphaenid** | **Exocoetid** | **Nomeid** | **Ommastrephid** | **Phrosinid** | **Scombrid** | **Serranid** | **Stomatopod** |
| --- | --- | --- | --- | --- | --- | --- | --- | --- | --- | --- |
| Sub-adult | Spring*Summer | 30.4% | 1.9% | 11.3% | 1.3% | 11.7% | 3.6% | 4.4% | 8.1% | 6.3% |
|  | Spring*Fall | 17.5% | 2.1% | 14.5% | 1.6% | 13.4% | 4.9% | 2.3% | 6.7% | 4.9% |
|  | Spring*Winter | 9.5% | 2.4% | 13.1% | 6.6% | 12.1% | 13.5% | 2.6% | 6.7% | 0.8% |
|  | Summer*Fall | 33.3% | 0.1% | 11.7% | 0.4% | 7.0% | 2.4% | 2.8% | 2.6% | 10.6% |
|  | Summer*Winter | 30.2% | 0.5% | 7.9% | 6.0% | 4.1% | 13.5% | 2.7% | 2.3% | 6.8% |
|  | Fall*Winter | 19.5% | 0.6% | 12.0% | 6.2% | 6.2% | 13.2% | 0.3% | 0.0% | 5.5% |
| Adult | Spring*Summer | 21.0% | 0.8% | 12.4% | 1.6% | 14.5% | 1.6% | 16.3% | 8.2% | 1.0% |
|  | Spring*Fall | 15.4% | 0.7% | 15.6% | 2.6% | 14.1% | 1.6% | 3.6% | 6.6% | 0.3% |
|  | Spring*Winter | 8.3% | 14.2% | 13.8% | 3.4% | 14.5% | 4.2% | 0.2% | 6.3% | 0.5% |
|  | Summer*Fall | 24.0% | 0.1% | 8.7% | 2.8% | 6.6% | 0.3% | 18.2% | 2.7% | 1.3% |
|  | Summer*Winter | 20.3% | 13.7% | 5.4% | 3.5% | 5.7% | 3.3% | 15.5% | 2.5% | 1.3% |
|  | Fall*Winter | 14.9% | 13.9% | 10.9% | 4.4% | 4.7% | 3.4% | 3.5% | 0% | 0.7% |
| Sub-adult*Adult | Spring | 7.6% | 2.9% | 18.0% | 2.2% | 18.5% | 4.9% | 2.6% | 12.2% | 0% |
|  | Summer | 32.6% | 0.1% | 3.8% | 1.0% | 7.2% | 0.2% | 21.0% | 5.7% | 8.5% |
|  | Fall | 22.1% | 0.1% | 13.6% | 2.3% | 6.8% | 2.2% | 3.6% | 0% | 5.3% |
|  | Winter | 10.9% | 14.5% | 9.5% | 7.8% | 4.1% | 13.5% | 0.5% | 0% | 1.2% |

**Supplemental Table S8**. The summary statistics (p-value and effective degrees of freedom) from generalized additive models (GAMs) examining seasonal patterns of prominent prey taxa (Figure 3) found in yellowfin tuna diets from the northern Gulf of Mexico (nGoM), in which p-values were significant for all models (p < 0.05).

| **Prey Taxa** | **p-value** | **edf** |
| --- | --- | --- |
| Exocoetids | 0.000027 *** | 3.688 |
| Serranids | < 2e-16 *** | 3.323 |
| Ommastrephids | 0.000000389 *** | 3.398 |
| Scombrids | < 2e-16 *** | 3.459 |
| Carangids | < 2e-16 *** | 3.529 |
| Stomatopods | 0.00000389 *** | 3.720 |
| Mugilids | 0.0154 * | 2.121 |
| Enoploteuthids | < 2e-16 *** | 2.648 |
| Brachyscelids | 0.00193 ** | 2.641 |
| Phrosinids | < 2e-16 *** | 3.251 |
| Nomeids | 0.00000177 *** | 3.146 |
| Salps | < 2e-16 *** | 3.149 |

**Supplemental Table S9.** The summary statistics (p-value and effective degrees of freedom) of hierarchical generalized additive models (HGAMs) for sub-adult and adult yellowfin tuna from the northern Gulf of Mexico (nGoM), where all seasonal trends for δ^13^C, δ^15^N, and δ^34^S values were significantly different (p < 0.05) with the exception of δ^13^C for adult yellowfin tuna.

| **Class** | **Isotope** | **p-value** | **edf** |
| --- | --- | --- | --- |
| Sub-adult | δ^13^C | 0.0000622 *** | 2.5726 |
|  | δ^15^N | 0.0000017 *** | 2.8018 |
|  | δ^34^S | 2E-16 *** | 3.4466 |
| Adult | δ^13^C | 0.327313 | 0.2391 |
|  | δ^15^N | 0.000428 *** | 2.9809 |
|  | δ^34^S | 0.0000147 *** | 2.8377 |
